# Zebrafish *gon4la* mutants recapitulate human *GON4L*-related growth disorders and reveal novel metabolic organs abnormalities

**DOI:** 10.1038/s41598-026-44674-3

**Published:** 2026-04-04

**Authors:** Su-Mei Tsai, Chia-Hao Hsu, I-Chieh Chiang, Wei-Neng Liao, Jen-Kun Chen, Yun-Jin Jiang

**Affiliations:** 1 Institute of Molecular and Genomic Medicine, National Health Research Institutes, Zhunan Township, Miaoli County Taiwan; 2 Institute of Molecular and Cellular Biology, National Taiwan University, Taipei, Taiwan; 3 Institute of Biomedical Engineering and Nanomedicine, National Health Research Institutes, Zhunan Township, Miaoli County Taiwan; 4 Biotechnology Center, National Chung Hsing University, Taichung, Taiwan; 5 Department of Life Science, Tunghai University, Taichung, Taiwan

**Keywords:** GON4L, Dwarfism, GH-IGF1 axis, Metabolic organs, Homeostasis, Cell biology, Developmental biology, Genetics, Molecular biology

## Abstract

Biallelic mutations in human *GON4L* have been linked to short stature, craniofacial abnormalities, and microcephaly, yet the underlying mechanisms remain unclear. GON4L is a nuclear protein composed of a YY1AP1-like region, two PAH domains, and one SANT domain, all implicated in transcriptional or chromatin regulation. Here, we define the function of Gon4la (a zebrafish ortholog of human GON4L) in post-embryonic growth and tissue homeostasis. We generated three *gon4la* mutant lines using CRISPR-Cas9, each predicted to express truncated proteins lacking the C-terminal PAH/SANT domains and differing in N-terminal YY1AP1-like region retention. All *gon4la* mutants displayed proportionate dwarfism with intestinal and pancreatic abnormalities, accompanied by expanded progenitor proliferation and impaired epithelial differentiation. Only mutants completely lacking the N-terminal YY1AP1-like region exhibited reduced *igf1a* expression, elevated growth hormone pathway activity, and increased mortality, consistent with endocrine dysregulation. Our findings demonstrate that distinct domains of Gon4la contribute differentially to systemic growth and metabolic organ homeostasis. Zebrafish *gon4la* mutants provide a tractable vertebrate model for dissecting mechanisms of *GON4L*-related developmental disorder, Li-Takada-Miyake syndrome (LTMS), and for functional testing of rare *GON4L* variants.

**Supplementary Information:**

The online version contains supplementary material available at 10.1038/s41598-026-44674-3.

## Introduction

Dwarfism, defined as short stature due to impaired growth, can be classified as disproportionate or proportionate. Disproportionate forms, such as achondroplasia caused by *FGFR3* mutations^1^, affect limb and trunk proportions, while proportionate dwarfism involves uniform growth retardation. Its causes are diverse, including genetic, hormonal, nutritional, and environmental factors. A key regulator of linear growth is growth hormone (GH), which stimulates hepatic production of insulin-like growth factor 1 (IGF1). Defects in this axis—such as growth hormone insensitivity (GHI)—commonly lead to dwarfism^2^. Beyond promoting height, GH/IGF1 signaling also supports tissue development and homeostasis by regulating cell proliferation and differentiation^3^.

Growth failure is a common pediatric concern with diverse genetic causes. Recent studies have identified mutations in chromatin regulatory proteins as an important mechanism underlying short stature, highlighting the importance of epigenetic control in systemic growth regulation. For example, the transcription factor Yin Yang 1 (YY1) interacts with chromatin remodeling complexes. Disruption of YY1 or its regulatory network leads to Gabriele-DeVries syndrome, which is characterized by intellectual disability and growth retardation^4,5^.

Among the proteins known to functionally interact with YY1, GON4L (Gon-4 Like) has emerged as a factor of particular interest due to accumulating evidence linking its dysfunction to severe growth disorders^6,7^. This conserved nuclear protein directly associates with YY1 via a distinct YY1-binding domain (YBD)^8,9^. In addition, GON4L also possesses two paired amphipathic helix (PAH) domains, and one Swi3, Ada2, N-Cor, and TFIIIB domain (SANT) domain^10^, which are implicated in mediating protein–protein interactions and chromatin association, respectively^11,12^. A GON4L paralogous protein, YY1-associated protein 1 (YY1AP1), carries an N-terminal YY1 binding domain (N-YBD), a distict transactivation domain (TD) and a C-terminal YY1 binding domain (C-YBD), further supporting the broader role of GON4L-related proteins in transcriptional regulation^13^. Collectively, these molecular features suggest that GON4L has the potential to influence fundamental developmental processes, including cell proliferation, differentiation, and systemic growth.

**Fig. 1:**
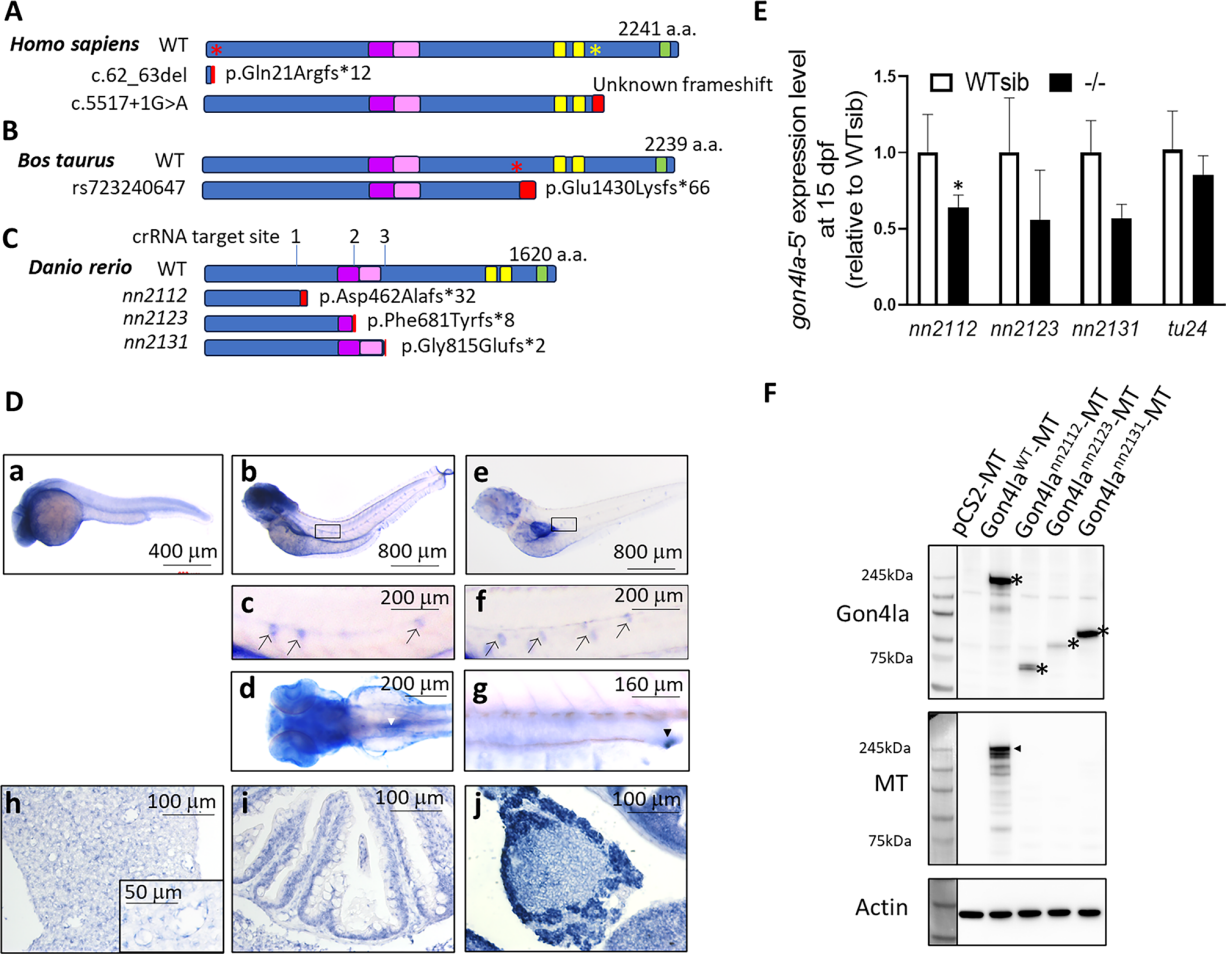
Evolutionary conservation, generation of mutant alleles, and spatiotemporal expression of zebrafish *gon4la*. (**A**–**C**) Schematic comparison of GON4L protein domains and clinical/natural variants in humans, cattle, and zebrafish. (**A**) WT and patient GON4L protein (predicted by SpliceAI). Red asterisk: premature stop codon resulted from a 2-bp deletion (c. 62_63del); yellow asterisk: canonical splice variant (c.5517+1G>A). Both variants were predicted to be subject to nonsense mediated RNA decay^6^. The c.5517+1G>A variant is predicted to disrupt splicing (e.g., leading to intron retention and frame shift of protein product). Due to lack of information, the abnormal transcript(s) in patients carrying c.5517+1G>A variant require experimental confirmation. (**B**) Wild type and predicted mutant cattle GON4L proteins. Red asterisk indicates premature stop codon resulting from the 1-bp deletion. (**C**) Zebrafish Gon4la protein sequences, showing three CRISPR/Cas9-targeted indels resulting in frameshift mutations and truncated proteins. Important domains are as indicated: purple box: N-terminal YY1-binding domain (N-YBD); pink box: putative transactivation domain (TD); yellow box: PAH domain; green box: SANT domain. Red box: additional peptide tail due to frameshift caused by introduced indels. (**D**, **a**–**g**) Spatiotemporal expression of *gon4la* mRNA via whole-mount in situ hybridization. (**a**) *gon4la* is strongly expressed in the brain at 1 dpf. At 3 (**b**, **c**) and 5 (**e**, **f**) dpf, *gon4la* is also detected in the neuromasts of the lateral lines (black arrows). Additionally, *gon4la* expression is observed in the gut looping region (**d**, dorsal view, white arrowhead) at 3 dpf and in the posterior gut and cloaca at 5 dpf (**g**, lateral view, black arrowhead). (**D**, **h**–**j**) Section in situ hybridization analysis of *gon4la* in adult tissues. (**h**) In the liver, *gon4la* is expressed in hepatocytes and sinusoidal endothelial cells (inset). (**i**) In the gut, *gon4la* is expressed in intestinal epithelial cells. (**j**) In the pancreas, *gon4la* is weakly expressed in islet cells and strongly expressed in exocrine cells. (**E**) qRT-PCR analysis of *gon4la* mRNA expression in WT siblings and *gon4la* homozygous mutants at 15 dpf. For comparison, *gon4lb*^*tu24*^ WT siblings and homozygous mutants at 5 dpf larvae were genotyped and analyzed. *, *p* < 0.05; *n* = 6. (**F**) Protein products expressed from WT and mutant *gon4la* constructs were detected by Western blot. Full-length Gon4la is detected in WT samples (asterisk: N-terminal; arrowhead: C-terminal Myc-tag, denoted as MT). Mutant alleles produce truncated proteins lacking the Myc tag. Actin was used as loading controls.

In humans, biallelic mutations in *GON4L* gene, leading to loss of the GON4L protein, have been identified as Li-Takada-Miyake syndrome (LTMS, phenotype MIM number #621212), which is associated with growth impairment affecting both prenatal and postnatal development. Affected individuals typically present prenatal-onset microcephaly, craniofacial abnormalities and postnatal short stature^6^. The identified variants in human patients are predicted to cause nonsense-mediated mRNA decay, leading to the loss of *GON4L* expression at both mRNA and protein levels (Fig. 1A). In cattle, a naturally occurred one-base pair deletion in *GON4L* leads to intron retention and a trucated protein lacking the PAH and SANT domains (Fig. 1B), which results in proportionate dwarfism in cattle^7^. While the observed phenotypes may vary, the consistent growth impairment across species when the GON4L protein is compromised suggests a conserved role for this protein in growth regulation.

Zebrafish are well-established for investigating conserved growth pathways, with genetic models recapitulating disorders such as Laron syndrome and Meier–Gorlin syndrome^14,15^. As clinical sequencing identifies rare *GON4L* variants in individuals with idiopathic growth failure, in vivo functional studies are urgently needed to determine their pathogenicity and mechanisms. In zebrafish genome, two *gon4l* paralogs exist: *gon4la* (XM_003200603.6, predicted sequence by automated computational analysis) and *gon4lb* (NM_001201535.1). Loss-of-function mutations in *gon4lb* cause severe developmental defects, including impaired somite and craniofacial development, apoptosis, and hematopoietic failure, resulting in early embryonic lethality^16–19^. While some of these phenotypes can be rescued by human *GON4L* mRNA^6^, the early lethality of *gon4lb* mutants precludes analysis of post-embryonic growth. The second zebrafish paralog, *gon4la*, has not been studied but offers a potential to investigate the post-embryonic functions of *GON4L* relevant to human diseases.

In the present study, we cloned zebrafish *gon4la* and investigated the role of Gon4la protein in post-embryonic growth and the maintenance of systemic metabolic tissues in zebrafish. Sequence conservation analysis (Hsu et al., submitted) further predicts a YY1AP1-like region (N-YBD and TD) in Gon4la. Therefore, to probe the contributions of distinct domains, we generated three *gon4la* mutant lines, which are expected to express proteins with varying configurations of the N-YBD and TD, while all lack the C-terminal PAH and SANT domains (Fig. 1C). We found that homozygous *gon4la* mutants, although viable into adulthood, exhibit reduced survival and proportionate dwarfism—mirroring phenotypes seen in humans and cattle with GON4L protein deficiency. In contrast to GON4L-deficient mice and zebrafish *gon4lb* mutants^16,19^, which are embryonically lethal, zebrafish *gon4la* mutants survive into adulthood. This viability, coupled with the differential protein truncations across the mutant lines, enables tissue-specific and stage-specific analyses of how the loss of specific Gon4la domains impacts growth and organ homeostasis at later time points. This model not only recapitulates the conserved growth phenotype but also reveals previously unrecognized abnormalities in metabolic organs, including the intestine, liver, and pancreas—tissues where *GON4L* mRNA is moderately expressed in humans. Taken together, these features make the zebrafish *gon4la* mutants a uniquely tractable in vivo platform for dissecting the domain-specific roles of GON4L protein and for evaluating rare human diseases associated with idiopathic short stature.

## Results

### *gon4la* is expressed in key metabolic organs of zebrafish

Zebrafish possess two GON4L homologs, *gon4la* and *gon4lb*, but only *gon4lb* has been functionally characterized. To investigate the potential role of *gon4la*, we first examined its expression pattern. *gon4la* mRNA was detected in the brain, neuromasts of the lateral line, and gut at 3 and 5 dpf (Fig. 1D, a–g) and in adult tissues, including the liver, intestine, and pancreas (Fig. 1D, h–j). Notably, robust expression was detected in intestinal epithelial cells and the exocrine pancreas.

### Generation and characterization of* gon4la* mutant lines

Three *gon4la* mutant lines (*gon4la*^*nn2112*^, *gon4la*^*nn2123*^, and *gon4la*^*nn2131*^) were generated via CRISPR/Cas9 (Fig. 1C). To exclude alternative splicing events that might restore the reading frame, we performed cDNA cloning and sequencing, which confirmed that all mutant transcripts retained the expected indel-induced frameshifts without evidence of exon skipping or in-frame splice variants (Table 1 and Supplementary Fig. 1). Notably, qRT-PCR analysis revealed a generalized downward trend in *gon4la* transcript levels across all mutant lines, likely due to the action of nonsense-mediated mRNA decay (NMD); however, this reduction was only statistically significant in the *gon4la*^*nn2112*^ allele (Fig. 1E). This suggests that while mRNA stability is affected, mutant protein production may not be completely abolished. These mutations result in premature stop codons that are predicted to yield truncated Gon4la proteins lacking the C-terminal PAH and SANT domains. Crucially, the *gon4la*^*nn2112*^ allele is further distinguished by the loss of the N-YBD and its associated putative transactivation domain (TD), while the *gon4la*^*nn2123*^ and *gon4la*^*nn2131*^ alleles are predicted to retain partial N-YBD and N-YBD/TD regions, respectively. Although available antibodies were insufficient for detecting endogenous proteins via Western blot in zebrafish tissue lysates, we utilized an in vitro heterologous expression system as a proof-of-principle to confirm that these mutant proteins can be translated (Fig. 1F). Antibody specificity for Gon4la was validated (Supplementary Fig. 2).

**Table 1:**
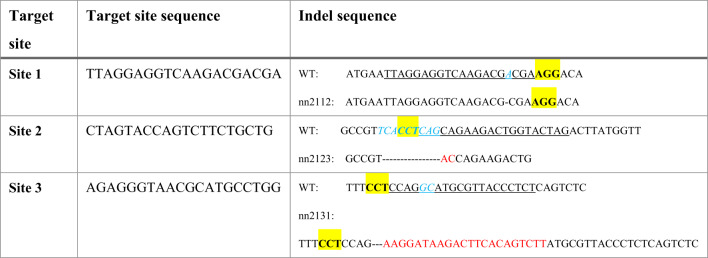
crRNA target sites and identified indel sequences.

Underline: target site; yellow highlight: PAM sequence; blue: deleted sequences in mutant alleles; red: inserted sequences in mutant alleles. Specific crRNAs were designed to target three distinct sites within the *gon4la* gene. Site 1: N-terminal to the N-YBD encoding region (aiming to disrupt all major domains); site 2: within the N-YBD encoding region (aiming to retain N-YBD but disrupt TD and C-terminal PAH and SANT domains); site 3: C-terminal to the TD but N-terminal to the PAH domains (aiming to retain N-YBD/TD but disrupt C-terminal PAH and SANT domains). Schematic illustration is depicted in Supplementary Fig. 1B.

### Delayed somatic and craniofacial development in *gon4la* mutants

**Fig. 2:**
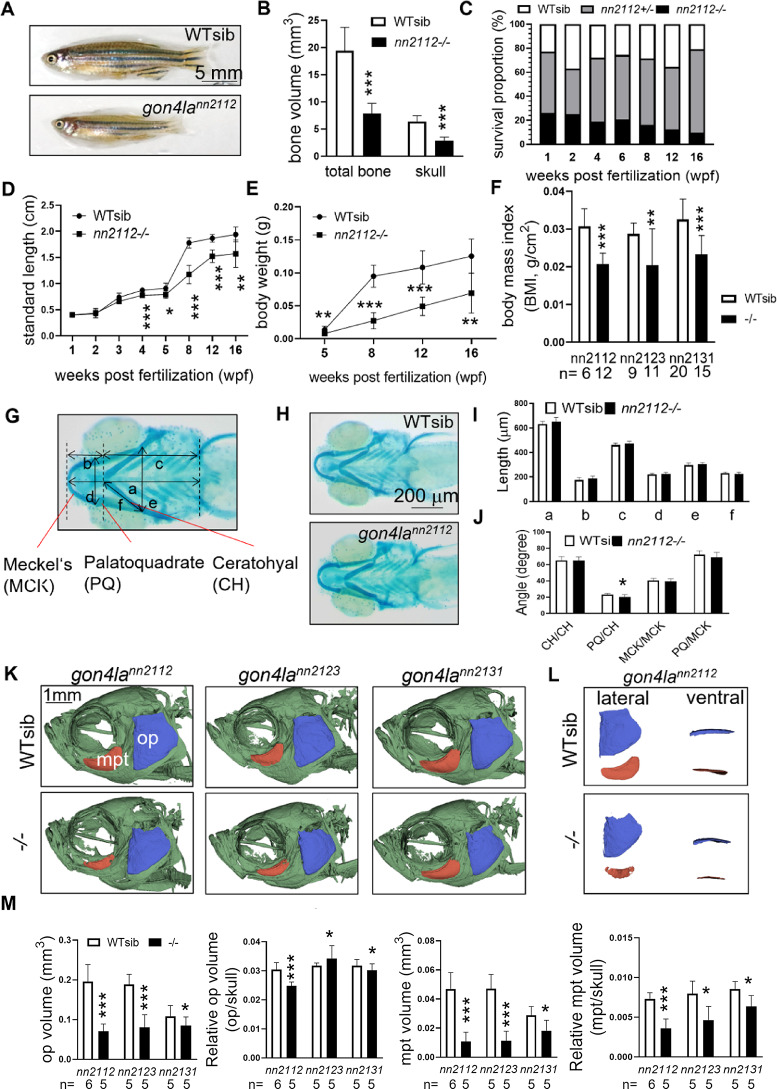
*gon4la* mutants show impaired growth and delayed craniofacial development. (**A**) Reduced body size and (**B**) significantly smaller total bone and skull volume in *gon4la*^*nn2112*^ mutants (3 mpf, n = 5/group). (**C**) Survival proportions of *gon4la*^*nn2112*^ homozygotes begin to decline after 12 wpf (Genotyping n ≥ 96/time point). (**D**, **E**) Growth metrics (length and weight) show delayed growth post-8 wpf (n ranges from 10/4 at 1 wpf to 11/6 at 16 wpf for WT/homo). (**F**) Comparison of body mass index (BMI) across all three mutant lines, showing a systemic reduction in body mass relative to length. (**G**, **H**) Craniofacial bone structure of WT siblings and *gon4la*^*nn2112*^ mutant larvae stained with Alcian blue at 5 dpf. (**I**) Quantification of six distinct length measurements (**a**–**f**), including total head length, ceratohyal cartilage length, and various joint distances, as indicated in (**G**). (**J**) Quantification of four key craniofacial cartilage angles: ceratohyal cartilages (CH/CH), palatoquadrate and ceratohyal (PQ/CH), Meckel’s cartilages (MCK/MCK), and palatoquadrate and Meckel’s cartilage (PQ/MCK) (*n* = 9–10/group). (**K**) µCT images of 3–4 mpf skulls (lateral view): Opercle (op, blue) and metapterygoid (mpt, red) are segmented and labeled. (**L**) Representative lateral and ventral views of segmented op and mpt from WT siblings and *gon4la*^*nn2112*^ mutants. (**M**) The sizes of op and mpt and their ratios to skull size were analyzed in all three mutant lines (*n* = 5–6/group).

All the *gon4la* mutant lines exhibited reduced overall body and head sizes in the juvenile and adult stages (Fig. 2A–B and Supplementary Fig. 3A). Among these three alleles, *gon4la*^*nn2112*^ exhibited the lowest survival rate (Supplementary Fig. 3B). Survival proportion began to decline after 8 weeks post-fertilization (wpf), with *gon4la*^*nn2112*^ homozygotes exhibiting the most conspicuous mortality by adulthood (Fig. 2C). In *gon4la*^*nn2112*^ mutants, body length remained comparable to those of WTs at 3 wpf but began to diverge from 4 wpf onward, with the gap progressively widening by 5–8 wpf (Fig. 2D). Body weight followed a similar trend, remaining initially unchanged but showing a noticeable reduction by 8 wpf (Fig. 2E), which indicates a post-larval onset of growth delay across multiple body size metrics. Consistent with this finding, quantitative analysis revealed significantly lower BMI values across all the homozygous mutants at 3–4 months post-fertilization (mpf) (Fig. 2F). Gonads were underdeveloped at 3 mpf across all three mutant lines (Supplementary Fig. 3C, a-d), probably contributing to infertility. Additionally, bloodless gills with a pale appearance were observed in some homozygous individuals of these lines, especially after 3 to 4 months of age (Supplementary Fig. 3C, e–f).

Craniofacial morphology appeared grossly normal during early development (Fig. 2G–J and Supplementary Fig. 4). However, craniofacial anomalies developed later according to micro-CT analysis, with reduced sizes of the opercle (op) and metapterygoid (mpt) at 3–4 mpf (Fig. 2K–L), and a relatively smaller mpt compared to skull size in all mutant lines (Fig. 2M), resembling juvenile-stage WT fish. These structures are critical components of the pharyngeal skeleton and play essential roles in jaw suspension, opercular movement, and feeding mechanics^20^. The size and shape of craniofacial elements such as the metapterygoid and opercle are commonly used as indicators of developmental progression in zebrafish^21^.

### *gon4la* mutant fish exhibit proportionate dwarfism phenotypes

**Fig. 3:**
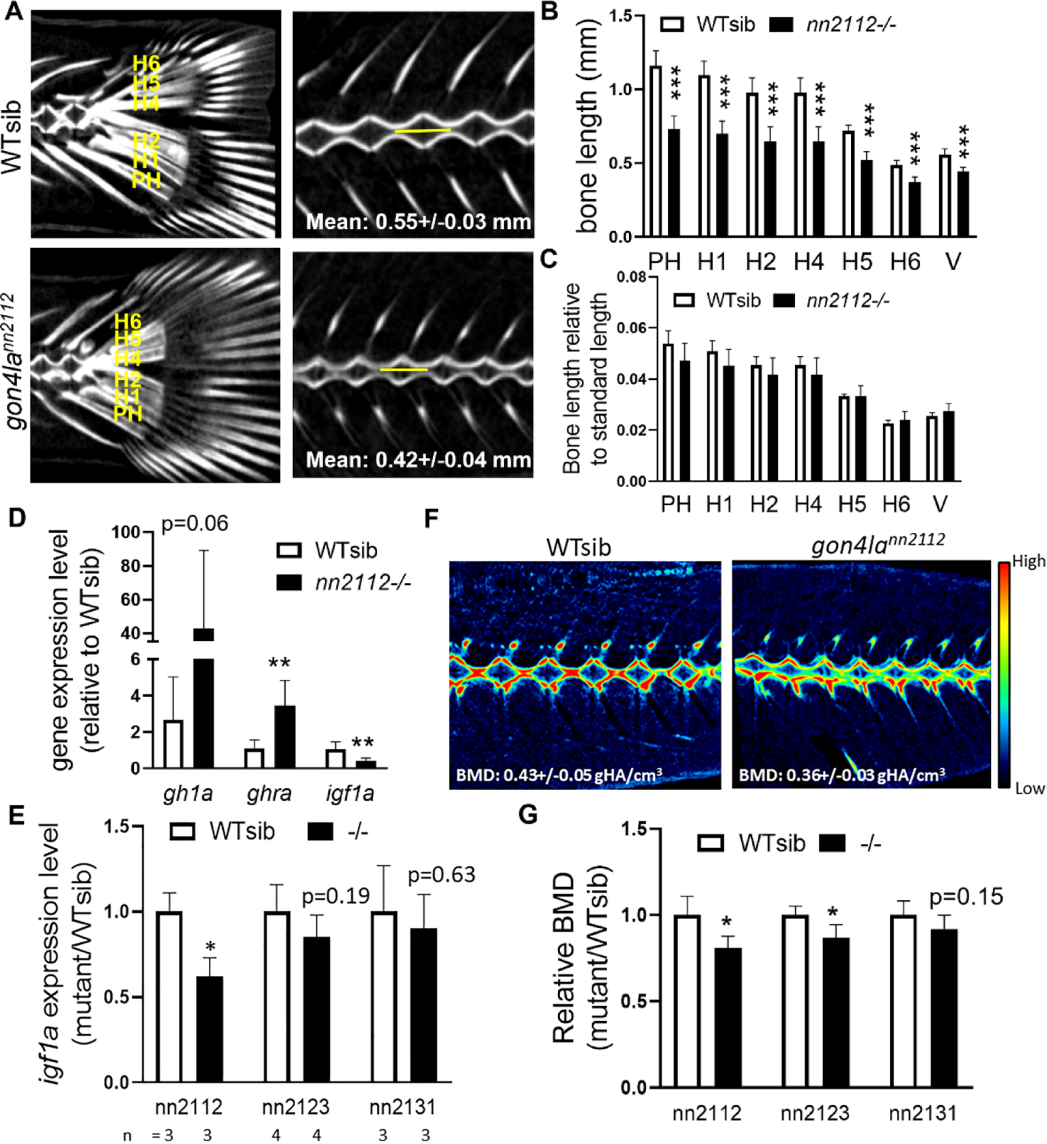
Allele-specific disruption of the GH/IGF-1 axis correlates with impaired bone mineralization. (**A**–**C**) µCT analysis of the caudal fin and vertebrae in adult WT and *gon4la*^*nn2112*^ mutants (3 mpf). (**A**) Representative images showing labeled endochondral bones (parhypural, PH; hypurals, H1–H6) and vertebral length (V, yellow lines). (**B**, **C**) Quantification of absolute bone lengths and relative ratios to standard length, demonstrating that *nn2112* mutants exhibit proportionate dwarfism (*n* = 5–6/group). (**D**) qRT-PCR analysis of GH/IGF-1 axis disruption (*gh1a*, *ghra*, *igf1a*) in *gon4la*^*nn2112*^. The significant reduction in hepatic *igf1a* is accompanied by compensatory upregulation of *gh1a* and *ghra*, characteristic of GH insensitivity (*n* = 6/group). (**E**) Comparative qRT-PCR analysis of hepatic *igf1a* expression across different *gon4la* mutant alleles. Note that *igf1a* levels are significantly compromised only in *nn2112*, while *nn2123* and *nn2131* show no statistically significant difference from WT siblings. Expression levels are normalized to that of *gapdh*. (**F**, **G**) Vertebral BMD is only significantly reduced in *gon4la*^*nn2112*^ (*n* = 5–6/group), which aligns with the loss of *igf1a* expression. Statistical analysis: *, *p* < 0.05; **, *p* < 0.01; ***, *p* < 0.001.

Dwarfism can be categorized as proportionate or disproportionate, reflecting distinct pathogenic mechanisms. To assess the nature of growth impairment in *gon4la* mutants, we analyzed the skeletal proportions via micro-CT. In the *gon4la*^*nn2112*^ mutants, both caudal fin bones and vertebrae were shortened; however, their ratios to standard length were preserved (Fig. 3A–C), indicating proportionate dwarfism. Similar analyses of *gon4la*^*nn2123*^ and *gon4la*^*nn2131*^ mutants revealed consistent shortening of these bones (Supplementary Fig. 5A–B). Interestingly, the caudal fin bones of *gon4la*^*nn2123*^ mutants appeared relatively longer when normalized to body length, though vertebral proportions remained comparable to WT siblings, as in the other two alleles (Supplementary Fig. 5C–D). Except for *gon4la*^*nn2131*^, *gon4la*^*nn2112*^* and gon4la*^*nn2123*^ mutants exhibited proportionally smaller skull volume and total bone volume (Supplementary Fig. 5E). Taken together, these findings demonstrate that Gon4la protein truncations lead to proportionate dwarfism.

### Allele-specific disruption of the somatotropic axis reveals a primary role for the N-terminal YBD in systemic growth regulation

Growth in zebrafish, mirroring mammals, is centrally regulated by the somatotropic axis, wherein growth hormone (GH) signals the liver to produce insulin-like growth factor 1a (Igf1a), driving somatic growth^22^. To dissect the role of Gon4la’s domains in this critical pathway, we compared hepatic *igf1a* expression across our three mutant alleles.

Critically, only the *gon4la*^*nn2112*^ allele, which results in a complete loss of all domains from the N-terminal YY1-binding domain (N-YBD) to PAH/SANT domains, showed a marked decrease in hepatic *igf1a* expression (Fig. 3D). This reduction was accompanied by classic features of GH insensitivity, including compensatory upregulation of pituitary *gh1a* and hepatic *ghra* expression (Fig. 3D). In contrast, *gon4la*^*nn2123*^ and *gon4la*^*nn2131*^ mutants, which retained partial N-YBD domain and complete N-YBD/TD domains, respectively, showed only a minor, statistically insignificant decrease in *igf1a* expression (Fig. 3E). This allele-specific effect indicates that the N-YBD (and not the C-terminal PAH/SANT domains) is the primary protein structure required for Gon4la to positively regulate the systemic Gh-Igf1a axis.

While all three alleles displayed proportionate dwarfism, bone mineral density (BMD) impairment was unique to the *gon4la*^*nn2112*^ and *gon4la*^*nn2123*^ lines; in contrast, the *gon4la*^*nn2131*^ allele maintained BMD at wild-type levels (Fig. 3F–G). These findings suggest a multi-layered regulatory model: while the C-terminal PAH/SANT domains are essential for overall somatic growth, the N-terminal YBD/TD region is specifically required to maintain the GH/Igf1a threshold necessary for normal bone mineralization. This distinction explains the observed discrepancy where *gon4la*^*nn2131*^ mutants remain significantly smaller yet maintain normal bone quality, further linking N-YBD-dependent endocrine function to skeletal health^23^.

### Hepatic deficits in ***gon4la***^***nn2112***^ mutants correlate with endocrine disruption

As the liver is a key source of circulating IGF-1 and a target of hormonal and nutritional regulation, we examined hepatic morphology in *gon4la* mutants. In *gon4la*^*nn2112*^ mutants, the livers exhibited enlarged sinusoidal spaces and reduced population of 2F11-positive biliary epithelial cells (Supplementary Fig. 6A–B). These abnormalities were exclusively observed in the *gon4la*^*nn2112*^ line and were absent in the other two mutant lines. Given the concurrent reduction in hepatic *igf1a* expression and decreased BMD, the liver abnormalities in *gon4la*^*nn2112*^ mutants may reflect a line-specific defect that contributes to its more pronounced growth impairment.

### Lack of PAH/SANT domains in Gon4la leads to gastrointestinal structural abnormalities

**Fig. 4:**
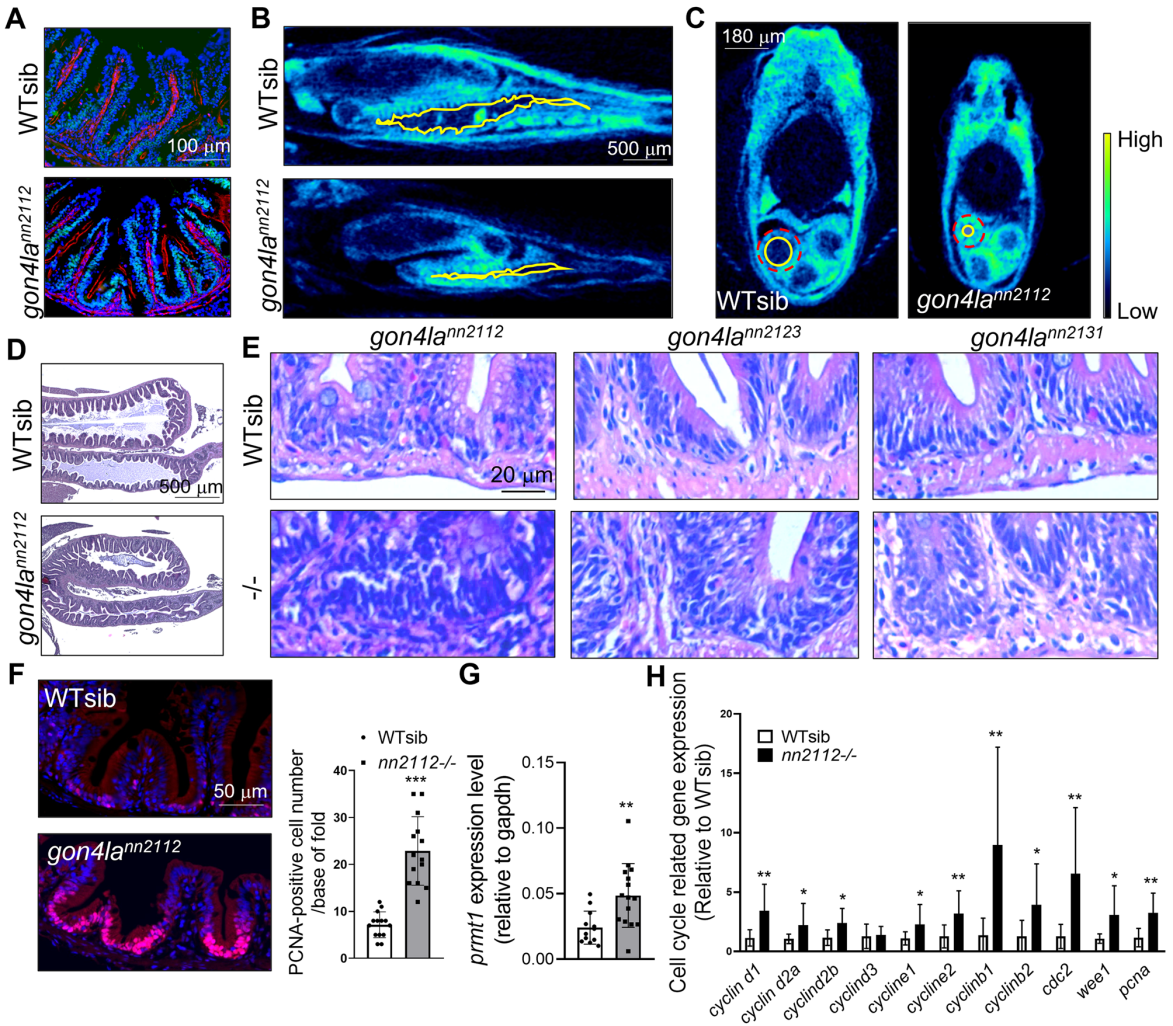
Disrupted protein localization, structural abnormalities, and hyperproliferation in the *gon4la* mutant intestine. (**A**) Immunofluorescent staining shows that WT Gon4la (green) is confined to intestinal fold bases; mutant protein is mislocalized towards apical regions. Actin (red), DAPI (blue). μCT images of (**B**) sagittal sections and (**C**) transverse sections of whole gut are taken from WT sibling and *gon4la*^*nn2112*^ mutant fish at 3–4 mpf. Posterior gut lumen was marked with yellow line. Dashed red line circled the posterior gut (*n* = 3/group). Relative lumen size of the posterior gut, calculated as the ratio of lumen area (yellow circle) to total transverse-sectional area (dashed red circle): WTsib: 0.375 ± 0.097; *gon4la*^*nn2112*^ mutant fish: 0.081 ± 0.060. *p* = 0.016. (**D**) H&E staining of gut highlights abnormal fold structures in *gon4la*^*nn2112*^ mutant fish. (**E**) H&E staining of posterior intestine shows densely packed nuclei at the fold bases across all three mutant lines. (**F**) PCNA immunostaining shows increased proliferating cells at fold bases in *gon4la*^*nn2112*^ mutants. Quantified across 14 folds of 3 fish per group. (**G**) qRT-PCR analysis shows upregulation of the ISC/TA cell marker *prmt1* in the intestine of *gon4la*^*nn2112*^ mutant fish. *n* = 12 for WT siblings and *n* = 15 for *gon4la*^*nn2112*^ mutants. *gapdh* was used as an internal control. (**H**) The expression of cell cycle-related markers, especially those associated with G2 phase, was significantly upregulated in the gut of *gon4la*^*nn2112*^ mutants relative to WT siblings, as determined by qRT-PCR. Data were normalized (relative to *gapdh* level) and expressed as fold change compared to WT controls (WT, *n* = 12; mutants, *n* = 14).

Given the independence of the *igf1a* axis in the *gon4la*^*nn2131*^ allele, we investigated whether Gon4la also contributed to the maintenance of local tissue homeostasis, particularly in the intestine, which is vital for nutrient absorption. In WT fish, the Gon4la protein was localized specifically to the interfold base of the intestinal epithelium (Fig. 4A, upper panel). However, all three mutant lines, including the *gon4la*^*nn2131*^ allele (which has normal *igf1a* expression), displayed mislocalization of the truncated protein to the apical epithelial regions (Fig. 4A and Supplementary Fig. 7A, lower panels). Furthermore, all three mutants showed reduced intestinal lumen diameter and abnormal fold architecture as revealed by micro-CT and histological examinations (Fig. 4B–D and Supplementary Fig. 7B). The presence of this shared intestinal phenotype in the *gon4la*^*nn2131*^ mutant strongly argues against it being a secondary effect of endocrine dysfunction. Instead, it supports a C-terminal domains-dependent structure and maintenance role for Gon4la in the gastrointestinal tract.

### Intestinal homeostasis is disrupted across all *gon4la* mutant fish

**Fig. 5:**
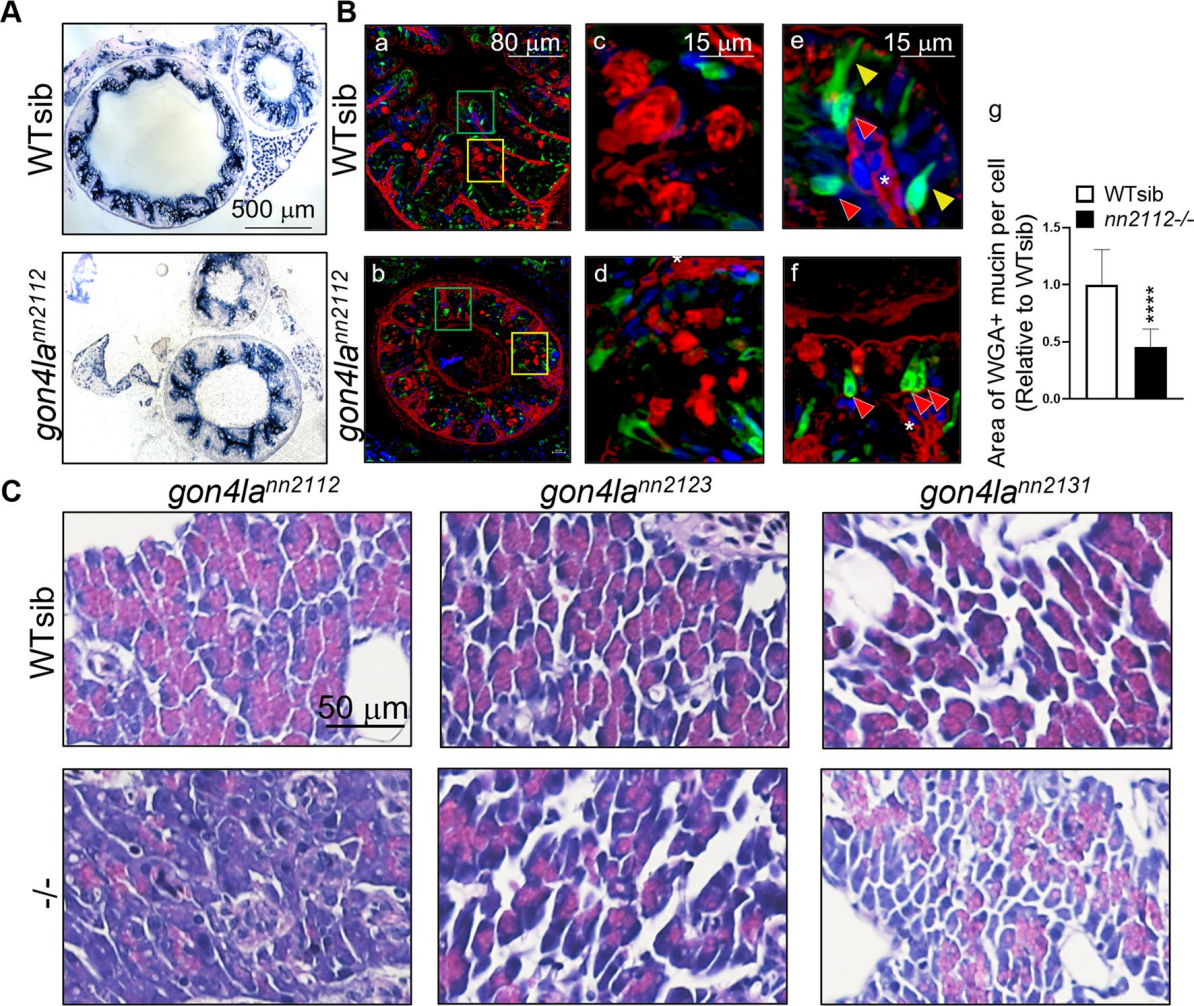
Impaired intestinal epithelial differentiation and pancreatic exocrine defects in *gon4la* mutants. (**A**) Alkaline phosphatase (AP) staining for enterocyte differentiation. (**B**) Immunofluorescent staining for goblet cells and enteroendocrine cells. (**a**, **b**) Cross section of posterior intestine at lower magnification. (**c**, **d**) Magnified views (from yellow squares) of goblet cells stained with WGA (red signals). (**e**, **f**) Magnified views (from green squares) of enteroendocrine cells (green signals, 2F11 antibody). Yellow arrowheads indicate matured enteroendocrine cells exhibited polarized morphology. Red arrowheads indicate less matured enteroendocrine cells. (**g**) WGA-positive mucin area within the goblet cell theca, shown as a ratio relative to WT siblings. Measurements were taken from *n* = 73 thecae from 4 WT sibling fish and *n* = 49 thecae from 4 *gon4la*^*nn2112*^ homozygous mutant fish. ****P < 0.0001. Quantification of the WGA-positive mucin area (theca size) relative to WT siblings shows significantly reduced secretory capacity in mutants. (**C**) H&E staining of pancreatic exocrine in WT and *gon4la* mutant fish. H&E staining of exocrine pancreas from adult WT and mutant fish at 3–4 mpf. Acinar cells in all mutant lines show marked reduction of zymogen granules.

To investigate whether structural changes were accompanied by altered epithelial dynamics, we analyzed intestinal cell proliferation and differentiation. All three *gon4la* mutant lines, regardless of their *igf1a* status, showed increased nuclear density at fold bases (Fig. 4E) and expanded proliferative zones (Fig. 4F and Supplementary Fig. 7A, upper panels). The importance of intestinal stem cells (ISCs) in maintaining intestinal homeostasis is well established^24^. In *gon4laⁿⁿ*^*2112*^ mutants, the expression levels of the ISC and transit amplifying cell (TA) marker *prmt1*^25,26^ and G2/M phase cell cycle genes were upregulated (Fig. 4G–H), suggesting dysregulated stem/progenitor proliferation. While enterocyte differentiation remained largely intact (Fig. 5A), terminal differentiation of secretory lineages were disrupted across all lines (Fig. 5B and Supplementary Fig. 7C): goblet cells contained less mucin within the theca (Fig. 5B: c,d and g), and enteroendocrine cells exhibited abnormal rounded shape (Fig. 5B: e–f). These findings indicate that the C-terminal PAH/SANT domains are required for intestinal progenitor control and epithelial maturation, defining a role in local metabolic tissue integrity.

### Pancreatic exocrine deficits parallel intestinal phenotypes, supporting PAH/SANT’s unified role

Given the shared endodermal origin of the intestine and pancreas, we hypothesized that the C-terminal PAH/SANT domains might regulate both tissues similarly. In WT fish, acinar cells were filled with dense zymogen granules (Fig. 5C, upper panels). These granules were markedly reduced or absent across all the *gon4la* mutant lines, including the *gon4la*^*nn2131*^ allele mutant (which has normal *igf1a*) (Fig. 5C, lower panels). These findings provide a second line of evidence that the C-terminal PAH/SANT domains are crucial for the maturation and function of endodermally-derived metabolic organs, independent of the N-YBD-mediated endocrine axis failure.

## Discussion

Zebrafish encode two *GON4L* paralogs, *gon4la* and *gon4lb*, which are likely derived from teleost genome duplication (Hsu et al., submitted). Although both paralogs show broad expression in the head (including eye and brain) during early development, their functional requirements diverge markedly across developmental stages. Consistent with this divergence, our data support a model of subfunctionalization following gene duplication, in which ancestral *GON4L* functions have been partitioned between the two paralogs. In this framework, *gon4lb* has retained an essential role during early embryogenesis^18,19^, whereas *gon4la* has acquired specialized functions in regulating intestinal epithelial integrity and post-embryonic somatic growth. Although *gon4la* is highly expressed maternally, its expression declines relative to *gon4lb* during mid-gastrulation (6–8 hpf), a critical developmental window that coincides with the onset of *gon4lb* essentiality for axis extendion (Supplementary Fig. 8A). Rather than indicating functional redundancy, this temporal divergence suggests that *gon4lb* becomes the predominant paralog expressed in specific embryonic contexts during this period. We propose that the lower relative abundance of *gon4la* during this period falls below the functional threshold required to support early embryonic development, explaining why *gon4la* cannot compensate for the loss of *gon4lb* and results in the lethality observed in *gon4lb* mutants. Importantly, this subfunctionalization is context-dependent, involving both spatial and dosage factors. *gon4lb* appears to provide sufficient compensatory support for *gon4la* in tissues or stages where their expression overlaps, which likely accounts for the viability and relatively mild craniofacial phenotypes of *gon4la* single mutants. However, such compensatory potential is not uniform across tissues. The non-redundant intestinal phenotypes in *gon4la* mutants likely arise from the tissue-specific enrichment of *gon4la* in the developing gut, where *gon4lb* expression is minimal or spatially restricted (Supplementary Fig. 8B). Thus, the divergent phenotypes of these two paralogs are dictated by a combination of their relative expression levels during early development and their distinct spatial domains during organogenesis.

While human *GON4L* mutations are generally predicted to cause disease through nonsense-mediated mRNA decay (NMD), the nature of the resulting proteins often remains unclear^6^. In zebrafish, incomplete elimination of premature termination codon–containing transcripts has been reported previously^27^, providing a biological context that mutant protein expression can occur. Our zebrafish model, which includes defined truncated Gon4la proteins, provides an opportunity to link domain loss with functional outcomes. Among the three alleles examined, only *gon4la*^*nn2112*^, which lacks the N-terminal YY1-binding domain (N-YBD) and the putative transactivation domain (TD), displayed reduced *igf1a* and increased *gh1a/ghra* expression, consistent with growth hormone (GH) insensitivity. This profile suggests a disrupted negative feedback loop where the lack of hepatic Igf1a leads to upregulation of pituitary *gh1a* and hepatic *ghra* but ultimately failing mechanism to restore Igf1a signaling. In contrast, alleles retaining partial or complete N-YBD/TD regions maintained near-normal *igf1a* expression despite dwarfism. These findings identify the N-YBD as a critical determinant of GH/IGF1 axis regulation, in agreement with the established role of YY1 in IGF1 transcription^28^. Nevertheless, the specific contribution of the TD remains unresolved and warrants future investigation.

In parallel, an *igf1a*-independent mechanism of dysfunction was evident in digestive organs. Intestinal and pancreatic abnormalities were consistently observed across all three alleles, regardless of N-terminal variations. Mutants exhibited narrowed intestinal lumens, impaired epithelial maturation, and reduced zymogen granules in pancreatic acinar cells, indicating an essential role for C-terminal regions of Gon4la in metabolic tissue homeostasis. The apparent phenomena of increased progenitor proliferation and reduced metabolic tissue integrity is best explained by a model of differentiation arrest. In the absence of Gon4la (specifically its C-terminal domains), progenitors fail to transit from a proliferative state into functionally mature lineages, leading to the observed defects in organ structure and a long-term depletion of secretory cells. Although goblet cell numbers were unchanged at 5 dpf (Supplementary Fig. 8C–D), differentiation defects became evident in adulthood (Fig. 5B and Supplementary Fig. 7C), pointing to impaired long-term epithelial maintenance or progressive depletion of secretory lineages.

Crucially, these defects were also present in the *gon4la*^*nn2131*^ allele, which retains intact N-YBD/TD domains and exhibits near-normal *igf1a* expression and bone mineral density. This persistent intestinal phenotype in *gon4la*^*nn2131*^ despite a near-normal GH/IGF-1 axis clearly dissociates local tissue differentiation defects from systemic IGF-1 levels. Therefore, we propose that the intestinal defects arise primarily from local cellular differentiation arrest, which, in the *gon4la*^*nn2112*^ line, is further exacerbated by systemic GH/IGF-1 coupling. This observation supports the conclusion that the C-terminal PAH and SANT domains are key structural determinants of Gon4la function in maintaining metabolic tissue integrity. Beyond these domains, additional structural features may also contribute to metabolic regulation. The GON4L paralog YY1AP1 contains both N-YBD/TD and a C-terminal YY1-binding domain (C-YBD)^13^, raising the possibility that Gon4la harbors additional and as yet uncharacterized regions influencing intestinal development and systemic growth. Given that YY1 regulates intestinal stem cell renewal^29^, Gon4la may act through both N-YBD/TD-dependent interactions with YY1 and C-terminal regions to maintain epithelial homeostasis.

**Fig. 6:**
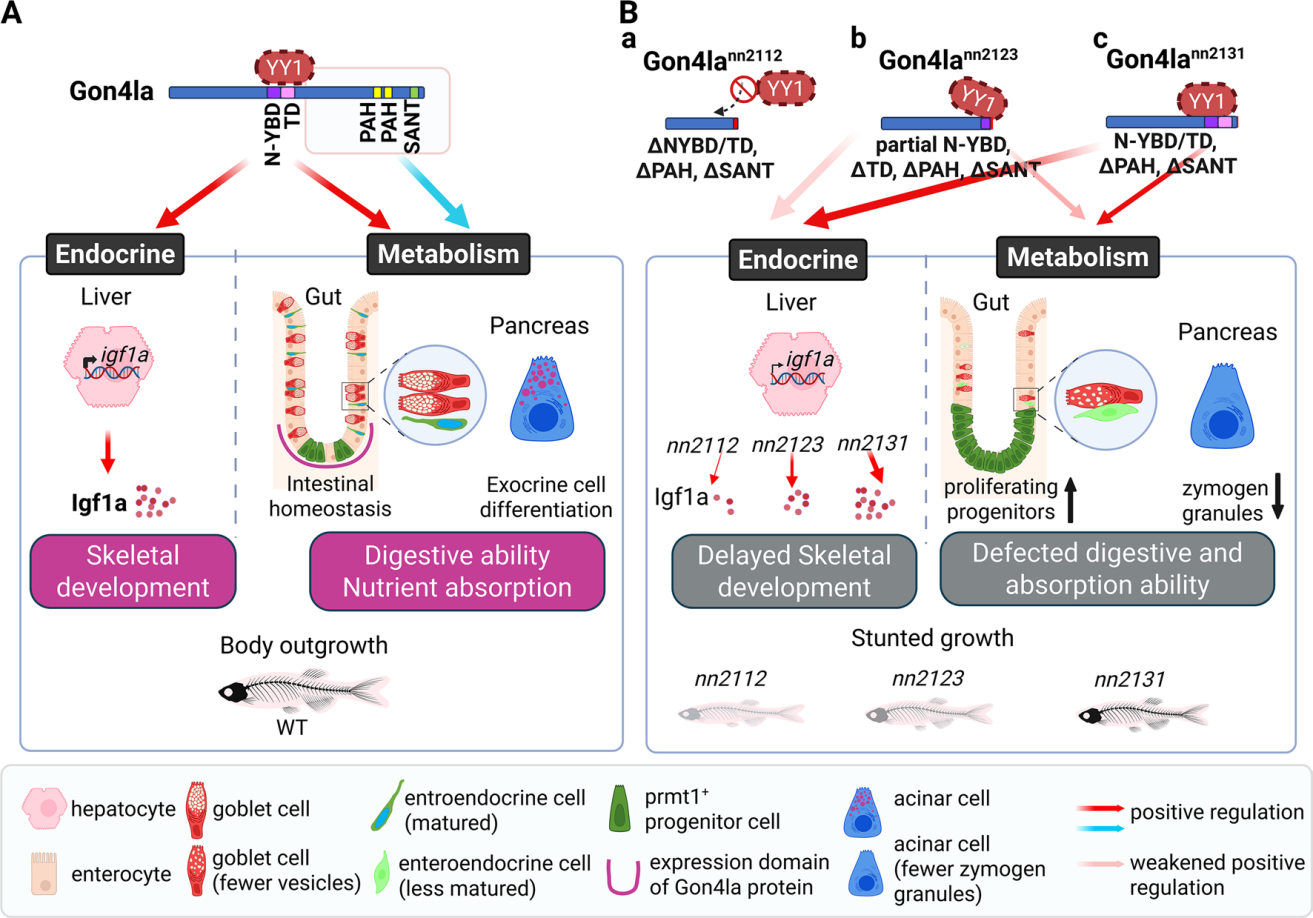
Proposed model illustrating domain specific functions of Gon4la in post-embryonic growth regulation. (**A**) WT scenario: Full-length WT Gon4la utilizes its N-terminal YY1-binding domain (N-YBD) and putative transactivation domain (TD) (solid red arrow) to regulate endocrine signaling (e.g., hepatic Igf1a production for skeletal development). Its C-terminal PAH and SANT domains (solid blue arrow) are crucial for metabolic organ homeostasis (e.g., intestinal integrity, pancreatic exocrine cell differentiation), collectively supporting normal post-embryonic growth. (**B**) *gon4la* mutant scenarios: (**a**) *gon4la*^*nn2112*^ (complete loss of N-YBD, TD, PAH, and SANT domains): this severe truncation prevents YY1 interaction and leads to impaired hepatic *igf1a* production, delayed skeletal development, and increased mortality; (**b**) *gon4la*^*nn2123*^ (retention of partial N-YBD but loss of TD, PAH, and SANT domains): this mutant shows an intermediate reduction in *igf1a* levels and skeletal abnormalities, potentially due to the retaining N-YBD capacity; (**c**) *gon4la*^*nn2131*^ (retention of N-YBD and TD but loss of PAH and SANT domains): this mutant exhibits the least severe endocrine disruption, with *igf1a* levels comparable to WT. Notably, the bone mineral density (BMD) is decreased in *gon4la*^*nn2112*^ (light gray skeleton) and *gon4la*^*nn2123*^ (gray skeleton), while BMD of *gon4la*^*nn2131*^ (black skeleton) remains comparable to WT. All three mutants lacking PAH/SANT domains, display significant intestinal and pancreatic abnormalities, including increased progenitor proliferation and defective differentiation. This underscores the role of these C-terminal domains in metabolic organ function. Because a mutant specifically lacking only the N-YBD or TD (while retaining PAH/SANT) was not generated, potential contributions of Gon4la’s N-YBD or TD to metabolic organ homeostasis (dotted black arrows) cannot be completely ruled out. Cellular phenotypes depicted: Goblet cells (matured vs. fewer vesicles), enteroendocrine cells (matured vs. less matured), *prmt1*^+^ progenitor cells, enterocytes, and acinar cells (normal vs. fewer zymogen granules). Expression domain of Gon4la protein in intestinal folds is indicated by the U-shaped magenta line. Arrow/Line key: Solid red arrow = positive regulation by N-YBD/TD on the endocrine system or metabolic organs; faint red arrow = weakened positive regulation; solid blue arrow = positive regulation by PAH/SANT domains on metabolism. This figure is created with BioRender.com.

Although GON4L-related metabolic abnormalities have not yet been reported in humans, its expression in liver, pancreas, and intestinal tissues^6,30^—combined with the phenotypes observed in zebrafish mutants—suggests that its role in metabolic regulation may be underrecognized. This translational relevance further supports the utility of *gon4la* mutant zebrafish for investigating how epithelial dysfunction contributes to systemic growth impairment. In summary, our study establishes *gon4la* mutant zebrafish as the first vertebrate model to functionally demonstrate a post-embryonic, multi-domain function of GON4L in growth regulation. As illustrated in our proposed model (Fig. 6), the allelic series reveal functional segregation: within Gon4la, in which the N-terminal YBD/TD region is critically linked to endocrine signaling, particularly the GH/IGF1 axis, whereas protein structures of the N-YBD/TD and PAH/SANT domains are essential for the structural and functional integrity of metabolic organs.

Notably, *gon4la*^*nn2112*^ mutants exhibited a trend toward more severe intestinal disorganization than the other two alleles. Although this difference was not statistically evaluated, the data suggest that loss of N-YBD/TD may exacerbate phenotypes primarily driven by C-terminal deficiency of Gon4la. This likely reflects differences in transcript stability modulated by the nonsense-mediated mRNA decay (NMD) and the relative half-life of the resulting truncated proteins across the three alleles, or a fundamental requirement for the C-terminal PAH/SANT domains to facilitate the formation of stable, functional protein complexes. While the limited sensitivity of available Gon4la antibodies for Western blotting of zebrafish tissue lysates precluded precise in vivo quantification of these proteins, the consistent detection of truncated products via immunofluorescence, coupled with allele-specific mRNA levels, provides a basis for our allelic interpretation. Furthermore, the absence of overt dominant-negative interference in alleles retaining the N-YBD/TD suggests that these truncated proteins function primarily as hypomorphic variants. This may be attributed to the relatively low in vivo stability of the truncated proteins or the necessity of the C-terminal PAH/SANT domains for stable and functional complex formation. As mutants specifically lacking only the N-YBD/TD or only the PAH/SANT domains were not generated, the precise contribution of Gon4la’s individual domains to metabolic organ homeostasis awaits further experimental validation. Nevertheless, this zebrafish platform enables functional interpretation of rare human GON4L variants and provides a powerful framework for dissecting how epithelial dysfunction contributes to systemic growth impairment.

## Methods

### Zebrafish maintenance and experimental treatments

Zebrafish (*Danio rerio*) were maintained under standard conditions as previously described^31^. Embryos from the wild-type (WT), *gon4lb*^*tu24*^, *gon4la*^*nn2112*^, *gon4la*^*nn2123*^, and *gon4la*^*nn2131*^ lines were staged as described previously^32^. All animal experiments were carried out in accordance with the ARRIVE guidelines and approved by the NHRI Institutional Animal Care and Use Committee (NHRI-IACUC-106063-A, NHRI-IACUC-110132-AC1, and NHRI-IACUC-112019-A), and carried out in accordance with the approved guidelines. All experimental animals (mutants and WT siblings) were generated from heterozygous x heterozygous (het x het) crosses. To keep mutant lines, heterozygous mutant fish were crossed to WT/AB strain fish.

Fish were anesthetized with MS-222 (tricaine methanesulfonate) (0.02% w/v, buffered with sodium bicarbonate to a neutral pH) for fin clipping or measurement of body weight and picture taking. Fish were put back in fresh water for recovery. Animals were monitored until fully recovered from anesthesia and transferred to system tanks with circulating water. Fish with signs of distress or discomfort, such as abnormal swimming and reduced activity, were monitored for additional few days. For tissue collection, fish < 14 days post fertilization (5–14 dpf) were euthanized by placing them in an ice water bath, followed by immersion in an overdose of MS-222 (0.04% w/v). Adult fish were sacrificed using an overdose of MS-222 (0.04% w/v) followed by prolonged immersion for at least 30 min. Cessation of opercular movement was examined prior to tissue harvesting.

### Whole-mount and section in situ hybridization

A 2.5 kb *gon4la* N-terminal fragment was amplified from adult WT brain cDNA via following primers: forward: 5′-CGTGTGTTGGCGGGTGTGTGT-3′ and reverse: 5′-GGATCAGTGGCTTCGGTGGTCT-3′. This fragment spanning nucleotides from 32 to 2572 bp, which includes the 5′UTR and partial 5′-end of *gon4la* coding sequence. After pairwise alignment analysis, no significant similarity was found between sequences of *gon4la* probe fragment and *gon4lb* nucleotide sequence (NM_001201535.1). The PCR product was subsequently cloned and inserted into pJet1.2, and orientation-confirmed plasmids were linearized with XbaI. Antisense probes were synthesized via the MEGAscript T7 Transcription Kit (Thermo Fisher) and labeled with digoxigenin (Roche). To examine the expression patterns of *gon4lb* at 120 h post-fertilization (hpf)*,* an antisense probe for *gon4lb* was used^33^. In situ hybridization of embryos (24, 72, and 120 hpf) and adult frozen sections was performed as previously described^33^. For adult tissues, samples were fixed overnight, cryoprotected in 30% sucrose, embedded in Tissue-Tek® O.C.T., and sectioned (5–10 μm). Following proteinase K treatment and post-fixation, hybridization was carried out overnight at 65 °C in HYB + buffer (HYB with 5 mg/mL yeast tRNA and 50 µg/mL heparin). DIG-labeled probe detection was performed using anti-DIG antibody and NBT/BCIP substrate. Images were taken on a Zeiss Axiovision Imager A1 or Discovery V8.

### Generation of *gon4la* mutant lines via the CRISPR/Cas9 system

*Gon4la* mutant lines were generated via CRISPR/Cas9 RNPs (Dharmacon, Genescript) as described previously^34^. Specific crRNAs were designed to target three distinct sites in the *gon4la* gene: one is N-terminal to the N-YBD encoding region (aiming to disrupt all major domains), one is within the N-YBD encoding region (aiming for partial N-YBD disruption and subsequent loss of the putative TD), and one is C-terminal to the TD (aiming to retain N-YBD/TD but disrupt C-terminal PAH/SANT domains). The protospacer sequences are listed in Table 1. This experimental design was predicted to yield three mutant alleles. All resultant proteins would lack the C-terminal PAH and SANT domains, yet they would critically differ in their N-terminal regions. Specifically, the *gon4la*^*nn2112*^ allele was predicted to cause complete loss of both the N-YBD and the putative TD. The *gon4la*^*nn2123*^ allele was expected to produce a protein with only a partial N-YBD and also lacking the putative TD. In contrast, the *gon4la*^*nn2131*^ allele was predicted to retain both a complete N-YBD and the putative TD (schematically presented in Fig. 2C). Founder identification was performed via a high-resolution melting assay (HRMA) and Sanger sequencing. Genomic DNA was extracted from caudal fins via 50 mM NaOH and neutralized with 1 M Tris–HCl (pH 8.0). The primers used for genotyping are listed in Supplementary Table 1. Mutations in *nn2112* were further confirmed via SalI digestion, while *nn2123* and *nn2131* were resolved on 4% agarose gels.

### RNA extraction and qRT‒PCR analysis for gene expression

To analyze the *gon4la* mRNA expression level of each mutant line, WT and homozygous mutant fish were collected at 15 days post-fertilization (dpf). To analyze *gon4la* and *gon4lb* mRNA expression level during early development, 20 WT embryos were pooled as one sample, three samples were collected at each indicated time point (from 1-cell stage to 4-somite stage). Tissue-specific RNA was extracted from pooled samples (< 5 weeks) or individuals (> 5 weeks) via RNAzol (Invitrogen) or MN Nucleospin RNA mini kits (Macherey–Nagel). Because Igf1 exerts sexually dimorphic effects on zebrafish growth and metabolism, with males being more sensitive to *igf1a* deficiency^22^, we specifically dissected tissues from male fish for *gh, ghr* and *igf1* gene expression analysis. cDNA was synthesized from 1 µg of RNA with a GoScript Kit (Promega). qPCR was performed with SensiFAST SYBR Master Mix (Meridian Life Science) on a QuantStudio™ 5 system (Applied Biosystems). The reactions included at least three biological replicates. The primer sequences are provided in Supplementary Table 2. *gapdh* served as an internal control.

### Polyclonal antibody production specific for the zebrafish Gon4la

A rabbit polyclonal antibody against zebrafish Gon4la was generated (ABclonal) using the peptide CKVARAEEPEDISESRDVNVTE (aa 19–40) to target an N-terminal region that remains intact across all three frameshift variants. This ensures that the antibody’s affinity for the truncated protein is not compromised. Serum collected post-immunization was affinity purified for subsequent applications.

### Expression of WT and mutant Gon4la in cell lines

WT and mutant *gon4la* transcripts were cloned and inserted into pCS2-MT (with 6 × Myc) vectors via the BamHI/ClaI sites to generate pCS2-*gon4la*-MT constructs. The primers used for cloning and sequencing are listed in Supplementary Table 3. The complete coding sequence for the wild-type *gon4la* was deposited in the NCBI GenBank and the European Nucleotide Archive (ENA) databases. The corresponding accession numbers are PX525845 and ERP184456, respectively. The plasmids were transfected into 293 T or HeLa cells via Lipofectamine 3000 (Invitrogen). For Western blotting, 293 T cells were lysed 24 h post-transfection; 20 μg of protein was probed with anti-Gon4la (1:1,000), anti-Myc (1:10,000, Santa Cruz), and anti-β-Actin (1:5,000, Sigma Aldrich) antibodies. ECL signals were detected with a ChemiDoc MP (Bio-Rad). Full-length unprocessed images for all Western blots are available in Supplementary Fig. 2C. For immunofluorescence, HeLa cells were fixed with 4% PFA and stained with primary antibodies (1:200) and fluorescent secondary antibodies (1:200). Nuclei were counterstained with DAPI.

### Alcian blue staining for craniofacial development and intestinal goblet cell differentiation in zebrafish larvae

Larvae at 5 or 6 dpf were fixed in 4% PFA and bleached in 1.5% H_2_O_2_/1% KOH. After PBT washes, the samples were stained with Alcian blue (0.1% in 1% HCl and 70% ethanol) for 20 min and then destained with acidic ethanol. The final images were analyzed and taken in glycerol. Craniofacial measurements and goblet cell counting were subsequently performed using ImageJ software^35^.

### Assessment of survival and growth rates of *gon4la* mutants

Survival rates of different *gon4la* mutant lines were monitored from 2 to 7 months of age, following genotyping at 1 month. To assess survival proportions of each genotype in the offspring of intercrosses between *gon4la*^*nn2112*^ heterozygotes, genotyping was performed at multiple time points between 1 to 16 weeks of age. At each time point, 3–5 independent clutches of progeny were sampled, and the proportions of WT, heterozygous, and homozygous genotypes were determined.

To quantify the growth rate, standard length (snout tip to caudal fin base) and body weight were recorded individually; body mass index (BMI) was calculated as weight (g)/[length (cm)]^2^. Sample sizes are indicated in the figure legend.

### Micro-CT analysis

Fish were anesthetized and then fixed for imaging. Bone samples were embedded in Spurr resin or wrapped in Saran wrap. PTA-stained soft tissues were prepared as described previously^36^. Micro-CT imaging was conducted on a SkyScan 1276 high-resolution desktop micro-CT scanner (Bruker; Kontich, Belgium), The X-ray tube with a 0.5 mm Al filter was operated at 55 kVp and 145 µA, providing a 6 µm resolution. Bone mineral density (BMD) was quantified by a hydroxyapatite calibration curve that was prepared from images of a micro-CT hydroxyapatite (HA) phantom (HA content: 0–1200 mgHA/cm^3^; micro-CT HA D32, QRM GmbH, Germany). 3D rendering, process, and analysis of images, including registration of head, precaudal vertebrae, caudal vertebrae, and tail segments, were performed using PMOD software (Version 4.0, PMOD Technologies Ltd., Switzerland). Image segmentation for particular bones (e.g. opercle and metapterygoid) was performed by 3D Slicer (Version 5.8.1, The Slicer Community). Bone lengths (caudal fin endochondral bones, vertebrae) were measured via PMOD 4.0.

### Tissue fixation and sectioning

The liver, gut, pancreas, spleen, and gallbladder were harvested together at the indicated time points and fixed in 4% paraformaldehyde overnight at 4 °C. After fixation, the tissues were washed in PBS, dehydrated through 25%, 50%, and 75% ethanol, and processed for paraffin embedding. Serial 2–3 µm thick paraffin sections were cut and placed on polylysine-coated slides for histological and immunofluorescence staining.

### Hematoxylin and eosin (H&E) staining

Standard H&E staining was performed on paraffin sections for routine histological examination. The stained slides were mounted with Surgipath Sub-X mounting medium (Leica).

### Lectin staining of goblet cells

To visualize intestinal goblet cells, the paraffin sections were deparaffinized and permeabilized with PBST (0.3% Triton X-100 in PBS) and then washed three times with buffer B (10 mM HEPES, 0.15 M NaCl, pH 7.5). The sections were incubated overnight at 4 °C with rhodamine-conjugated wheat germ agglutinin (WGA; 1:100 in buffer B, Vector Lab). After washing with PBS, the slides were mounted with antifade DAPI mounting medium for fluorescence microscopy.

### Alkaline phosphatase staining of enterocytes

To detect intestinal enterocytes, freshly dissected tissues were snap frozen, and 10 μm-thick cryosections were prepared. The sections were air-dried, washed with PBS, and then incubated with NBT/BCIP in NTMT buffer (pH 9.5) to detect alkaline phosphatase activity. After color development, the sections were destained in methanol and mounted with glycerol for imaging.

### Immunofluorescence staining for detection of Gon4la, proliferation, and secretory markers

Serial 2 μm paraffin sections were used for immunofluorescence staining. The slides were deparaffinized, rehydrated in PBS, and subjected to antigen retrieval at 95 °C for 10 min. After cooling to room temperature, the sections were permeabilized with PBST (0.3% Triton X-100 in PBS) and blocked with 5% BSA. The following primary antibodies were diluted in blocking solution and incubated overnight at 4 °C: anti-Gon4la (ABclonal, 1:200), anti-PCNA (PC10, Cell Signaling, 1:1,000), and anti-FIS 2F11/2 (Abcam, 1:200) to label Gon4la, proliferating cells, and secretory epithelial cells (e.g., biliary and enteroendocrine cells), respectively. For the double labeling of goblet cells and enteroendocrine cells, rhodamine-labeled WGA was applied prior to BSA blocking. Following washes, appropriate Alexa Fluor-conjugated secondary antibodies (Invitrogen) were applied, and the sections were mounted with Diamond anti-fade DAPI mounting medium for fluorescent microscopic or confocal imaging. Quantification of the PCNA-positive cells or area of WGA-positive mucin within the thecae of goblet cells was performed using ImageJ software^35^.

## Supplementary Information

Below is the link to the electronic supplementary material.

Supplementary Material 1

Supplementary Material 2

Supplementary Material 3

Supplementary Material 4

## Data Availability

The authors confirm that the data supporting this study are available within the article. Original data that support the findings of this study are available from the corresponding author upon reasonable request. The *gon4la* sequence has been deposited in the NCBI GenBank (accession number: PX525845) and ENA (accession number: ERP184456).

## References

[1] Ornitz, D. M. & Legeai-Mallet, L. Achondroplasia: Development, pathogenesis, and therapy.. *Dev. Dyn.* **246**, 291–309. 10.1002/dvdy.24479 (2017). 27987249 10.1002/dvdy.24479 PMC5354942

[2] Mastromauro, C., Giannini, C. & Chiarelli, F. Short stature related to growth hormone insensitivity (GHI) in childhood. *Front. Endocrinol. (Lausanne)* **14**, 1141039. 10.3389/fendo.2023.1141039 (2023). 37008935 10.3389/fendo.2023.1141039 PMC10050683

[3] Dehkhoda, F., Lee, C. M. M., Medina, J. & Brooks, A. J. The growth hormone receptor: Mechanism of receptor activation, cell signaling, and physiological aspects. *Front. Endocrinol. (Lausanne)* **9**, 35. 10.3389/fendo.2018.00035 (2018). 29487568 10.3389/fendo.2018.00035 PMC5816795

[4] Gabriele, M. et al. YY1 haploinsufficiency causes an intellectual disability syndrome featuring transcriptional and chromatin dysfunction. *Am. J. Hum. Genet.* **100**, 907–925. 10.1016/j.ajhg.2017.05.006 (2017). 28575647 10.1016/j.ajhg.2017.05.006 PMC5473733

[5] Gabriele, M., Lopez Tobon, A., D’Agostino, G. & Testa, G. The chromatin basis of neurodevelopmental disorders: Rethinking dysfunction along the molecular and temporal axes.. *Prog. Neuropsychopharmacol. Biol. Psychiatry* **84**, 306–327. 10.1016/j.pnpbp.2017.12.013 (2018). 29309830 10.1016/j.pnpbp.2017.12.013

[6] Li, S. et al. Biallelic loss-of-function variants in GON4L cause microcephaly and brain structure abnormalities.. *NPJ Genom. Med.* **9**, 55. 10.1038/s41525-024-00437-5 (2024). 39500882 10.1038/s41525-024-00437-5 PMC11538285

[7] Schwarzenbacher, H. et al. A frameshift mutation in GON4L is associated with proportionate dwarfism in Fleckvieh cattle.. *Genet. Sel. Evol.* **48**, 25 (2016). 27036302 10.1186/s12711-016-0207-z PMC4818447

[8] Ohtomo, T., Horii, T., Nomizu, M., Suga, T. & Yamada, J. Molecular cloning of a structural homolog of YY1AP, a coactivator of the multifunctional transcription factor YY1. *Amino Acids* **33**, 645–652 (2007). 17297563 10.1007/s00726-006-0482-z

[9] Verheul, T. C. J., van Hijfte, L., Perenthaler, E. & Barakat, T. S. The why of YY1: Mechanisms of transcriptional regulation by Yin Yang 1.. *Front. Cell Dev. Biol.* **8**, 592164. 10.3389/fcell.2020.592164 (2020). 33102493 10.3389/fcell.2020.592164 PMC7554316

[10] Lu, P. et al. The developmental regulator protein Gon4l associates with protein YY1, co-repressor Sin3a, and Histone Deacetylase 1 and mediates transcriptional repression. *J. Biol. Chem.* **286**, 18311–18319 (2011). 21454521 10.1074/jbc.M110.133603 PMC3093903

[11] Spronk, C. A. et al. The Mad1-Sin3B interaction involves a novel helical fold. *Nat. Struct. Biol.* **7**, 1100–1104 (2000). 11101889 10.1038/81944

[12] Boyer, L. A. et al. Essential role for the SANT domain in the functioning of multiple chromatin remodeling enzymes.. *Mol. Cell* **10**, 935–942 (2002). 12419236 10.1016/s1097-2765(02)00634-2

[13] Wang, C. Y. et al. YY1AP, a novel co-activator of YY1. *J. Biol. Chem.* **279**, 17750–17755 (2004). 14744866 10.1074/jbc.M310532200

[14] McMenamin, S. K., Minchin, J. E., Gordon, T. N., Rawls, J. F. & Parichy, D. M. Dwarfism and increased adiposity in the gh1 mutant zebrafish Vizzini. *Endocrinology* **154**, 1476–1487. 10.1210/en.2012-1734 (2013). 23456361 10.1210/en.2012-1734 PMC3602633

[15] Yao, L., Chen, J., Wu, X., Jia, S. & Meng, A. Zebrafish cdc6 hypomorphic mutation causes Meier-Gorlin syndrome-like phenotype. *Hum. Mol. Genet.* **26**, 4168–4180. 10.1093/hmg/ddx305 (2017). 28985365 10.1093/hmg/ddx305 PMC5886151

[16] Colgan, D. F., Goodfellow, R. X. & Colgan, J. D. The transcriptional regulator GON4L is required for viability and hematopoiesis in mice. *Exp. Hematol.* **98**, 25–35. 10.1016/j.exphem.2021.04.001 (2021). 33864850 10.1016/j.exphem.2021.04.001 PMC8184620

[17] Williams, M. L. K. et al. Gon4l regulates notochord boundary formation and cell polarity underlying axis extension by repressing adhesion genes. *Nat. Commun.* **9**, 1319 (2018). 29615614 10.1038/s41467-018-03715-w PMC5882663

[18] Lim, C.-H., Chong, S.-W. & Jiang, Y.-J. Udu deficiency activates DNA damage checkpoint. *Mol. Biol. Cell* **20**, 4183–4193. 10.1091/mbc.E09-02-0109 (2009). 19656853 10.1091/mbc.E09-02-0109 PMC2754932

[19] Liu, Y. et al. The zebrafish *udu* gene encodes a novel nuclear factor and is essential for primitive erythroid cell development. *Blood* **110**, 99–106. 10.1182/blood-2006-11-059204 (2007). 17369489 10.1182/blood-2006-11-059204

[20] Cubbage, C. C. & Mabee, P. M. Development of the cranium and paired fins in the zebrafish *Danio rerio* (Ostariophysi, Cyprinidae). *J. Morphol.* **229**, 121–160. (1996). 29852585 10.1002/(SICI)1097-4687(199608)229:2<121::AID-JMOR1>3.0.CO;2-4

[21] Charles, J. F. et al. Utility of quantitative micro-computed tomographic analysis in zebrafish to define gene function during skeletogenesis. *Bone* **101**, 162–171. 10.1016/j.bone.2017.05.001 (2017). 28476577 10.1016/j.bone.2017.05.001 PMC5512604

[22] Zeng, N. et al. Sexual dimorphic effects of igf1 deficiency on metabolism in zebrafish. *Front. Endocrinol. (Lausanne)* **13**, 879962. 10.3389/fendo.2022.879962 (2022). 35966057 10.3389/fendo.2022.879962 PMC9372914

[23] Capozzi, A., Casa, S. D., Altieri, B. & Pontecorvi, A. Low bone mineral density in a growth hormone deficient (GHD) adolescent. *Clin. Cases Miner. Bone Metab.* **10**, 203–205 (2013). 24554933 PMC3917585

[24] Barker, N. Adult intestinal stem cells: Critical drivers of epithelial homeostasis and regeneration. *Nat. Rev. Mol. Cell Biol.* **15**, 19–33. 10.1038/nrm3721 (2014). 24326621 10.1038/nrm3721

[25] Peng, Z., Bao, L., Shi, B. & Shi, Y. B. Protein arginine methyltransferase 1 is required for the maintenance of adult small intestinal and colonic epithelial cell homeostasis. *Int. J. Biol. Sci.* **20**, 554–568. 10.7150/ijbs.89958 (2024). 38169732 10.7150/ijbs.89958 PMC10758107

[26] Tavakoli, S., Zhu, S. & Matsudaira, P. Cell clusters containing intestinal stem cells line, the zebrafish intestine intervillus pocket. *iScience* **25**, 104280. 10.1016/j.isci.2022.104280 (2022). 35586068 10.1016/j.isci.2022.104280 PMC9108511

[27] Anderson, J. L. et al. mRNA processing in mutant zebrafish lines generated by chemical and CRISPR-mediated mutagenesis produces unexpected transcripts that escape nonsense-mediated decay. *PLoS Genet.* **13**, e1007105. 10.1371/journal.pgen.1007105 (2017). 29161261 10.1371/journal.pgen.1007105 PMC5716581

[28] Blättler, S. M. et al. Yin Yang 1 deficiency in skeletal muscle protects against rapamycin-induced diabetic-like symptoms through activation of insulin/IGF signaling. *Cell Metab.* **15**, 505–517. 10.1016/j.cmet.2012.03.008 (2012). 22482732 10.1016/j.cmet.2012.03.008 PMC3324784

[29] Perekatt, A. O. et al. YY1 is indispensable for Lgr5+ intestinal stem cell renewal. *Proc. Natl. Acad. Sci. U S A* **111**, 7695–7700. 10.1073/pnas.1400128111 (2014). 24821761 10.1073/pnas.1400128111 PMC4040551

[30] Fagerberg, L. et al. Analysis of the human tissue-specific expression by genome-wide integration of transcriptomics and antibody-based proteomics. *Mol. Cell. Proteomics* **13**, 397–406. 10.1074/mcp.M113.035600 (2014). 24309898 10.1074/mcp.M113.035600 PMC3916642

[31] You, M.-S. et al. A sketch of the Taiwan Zebrafish Core Facility. *Zebrafish* **13**, S24–S29 (2016). 27267235 10.1089/zeb.2015.1208

[32] Kimmel, C. B., Ballard, W. W., Kimmel, S. R., Ullmann, B. & Schilling, T. F. Stages of embryonic development of the zebrafish. *Dev. Dyn.* **203**, 253–310 (1995). 8589427 10.1002/aja.1002030302

[33] Tsai, S. M., Chu, K. C. & Jiang, Y. J. Newly identified Gon4l/Udu-interacting proteins implicate novel functions. *Sci. Rep.* **10**, 14213. 10.1038/s41598-020-70855-9 (2020). 32848183 10.1038/s41598-020-70855-9 PMC7449961

[34] Hoshijima, K. et al. Highly efficient CRISPR-Cas9-based methods for generating deletion mutations and F0 embryos that lack gene function in zebrafish. *Dev. Cell* **51**, 645-657.e644. 10.1016/j.devcel.2019.10.004 (2019). 31708433 10.1016/j.devcel.2019.10.004 PMC6891219

[35] Schneider, C. A., Rasband, W. S. & Eliceiri, K. W. NIH Image to ImageJ: 25 years of image analysis. *Nat. Methods* **9**, 671–675. 10.1038/nmeth.2089 (2012). 22930834 10.1038/nmeth.2089 PMC5554542

[36] Ding, Y. et al. Computational 3D histological phenotyping of whole zebrafish by X-ray histotomography. *Elife*10.7554/eLife.44898 (2019). 31063133 10.7554/eLife.44898 PMC6559789

