# Supplementary material for: Zebrafish gon4la mutants recapitulate human GON4L-related growth disorders and reveal novel metabolic organs abnormalities
Source: Sci Rep. 2026 Apr 4;16:16357. doi: 10.1038/s41598-026-44674-3 (PMC13212936; doi:10.1038/s41598-026-44674-3)
Supplement: Supplementary file 1 — Supplementary Material 1 [file 41598_2026_44674_MOESM1_ESM.docx]

Supplementary Table 1. Primer pairs used for screening and genotyping of *gon4la* mutant lines.

| Mutant line | Sense (5’🡪3’) | Antisense (5’🡪3’) | Application |
| --- | --- | --- | --- |
| *nn2112* | CTCAATGCTGAACCTGATCT | CTGAATGTTCTCCAGAATGC | HRMA/ Genotyping |
|  | TACCCAGAATGCTTTTCACT | TTCAGGGTCATGGATGTTAT | Sequencing |
|  | GCTGAACCTGATCTCCATCACCTATAGTTTCAGGATGAATTAGGAGGTCAAGTCG | CGATAACGCTGCTTCAACACA T | Genotyping |
| *nn2123* | CCCAGAGCTACTTCCTACAA | TGTATAATGGTCGAGGTTGA | HRMA/  Genotyping |
|  | CAATGTCATCCAAAATGTGA | GCTGCAGCACTAAGTTTACC | Sequencing |
| *nn2131* | AGAACATGTAACCAGGCATC | GAAGCTTCAAGGGTCTGAG | HRMA/  Genotyping |
|  | CATGACATTAGACGGGTTTT | GCTTGGTTTAAAAGTGGTTG | Sequencing |
