# Supplementary material for: Zebrafish gon4la mutants recapitulate human GON4L-related growth disorders and reveal novel metabolic organs abnormalities
Source: Sci Rep. 2026 Apr 4;16:16357. doi: 10.1038/s41598-026-44674-3 (PMC13212936; doi:10.1038/s41598-026-44674-3)
Supplement: Supplementary file 2 — Supplementary Material 2 [file 41598_2026_44674_MOESM2_ESM.docx]

Supplementary Table 2. Primer pairs used for qRT-PCR analysis.

| Gene Name | Sense (5’🡪3’) | Antisense (5’🡪3’) |
| --- | --- | --- |
| *gapdh* | GTGGAGTCTACTGGTGTCTTC | GTGCAGGAGGCATTGCTTACA |
| *gon4la* | CGTGTGTTGGCGGGTGTGT | TCTTCTGCTCTCGCTACTTTACACTG |
| *gh1a* | GAAAGCCTCCGAAAACCAG | GCAGAACGACAGAGGGAAGA |
| *igf1a* | GTGGAGACAGGGGCTTTTATTT | CTTTGAAAGCAGCATTCGTC |
| *ghra* | TCCTCCTTCATCGCTGCCTAT | GCAAAGGCTGATAGAAAGGAAACA |
| *prmt1* | ATGGCGGAGACGGCAGAC | CACTTCCCACATCCAGCACAA C |
| *cyclin d1* | TGGGATCTGGCCTCAGTGAC | TGAAGTTGACGTCTGTCGCAC |
| *cyclin d2a* | AGCCGTATTAAAGGTCGAAAAGG | CCTCGCAGACCTCTAACATCCA |
| *cyclin d2b* | ACTGCTGTGGGAGTTGGTGG | AAGGTTTGCGTGTGCTTGCG |
| *cyclin d3* | CATCGCCCTCACGGCTACAG | ACATGCAGAGAACGCCTTGTCC |
| *cyclin e1* | TCAGGGCTGAAGTGGTGTGA | GGAGTGAACCTTTCCCAGCC |
| *cyclin e2* | GCACTGGACACTGCGGACAA | GGG ACT CTT CTA TTG CAC TCG CC |
| *cyclin b1* | GTACCCACCAGAGATTGCAG | GGTAGAGGCCTTCCAAAACC |
| *cyclin b2* | CTTAAGCCCATCATGCAGCT | GCTGCTTGCGTATTTCTTCC |
| *cdc2* | TGTACGCCTGCTAGATGTGC | TGATGGGATGGAGTCCAAAT |
| *wee1* | GAGCAAAATGCACTTCGTGA | TGCCCAGGCAGAATAATACC |
| *pcna* | GGCAACATCAAGCTCTCACA | TGCACTGGCTCATTCATCTC |
