# Supplementary material for: Zebrafish gon4la mutants recapitulate human GON4L-related growth disorders and reveal novel metabolic organs abnormalities
Source: Sci Rep. 2026 Apr 4;16:16357. doi: 10.1038/s41598-026-44674-3 (PMC13212936; doi:10.1038/s41598-026-44674-3)
Supplement: Supplementary file 3 — Supplementary Material 3 [file 41598_2026_44674_MOESM3_ESM.docx]

Supplementary Table 3. Primer used for cloning and sequencing *gon4la* constructs.

| Primer Name | Primer sequence (5’🡪3’) | Application |
| --- | --- | --- |
| *BamHI-gon4la-5'UTR-S* | CGGGATCCTGTGTATCTCGTCACCGCAGC | cloning |
| *ClaI-noSTOP-gon4la-AS* | CCATCGATACTGTGTCCAATTGTCTGGTGTGTG | cloning |
| *pJet-S* | CGACTCACTATAGGGAGAGCGGC | sequencing |
| *pJet-AS* | AAGAACATCGATTTTCCATGGCAG | sequencing |
| *gon4la-S1* | GCAAACGGAGACTGAATAAC | sequencing |
| *gon4la-S2* | TGAAAGCTGCTATCAGTGAA | sequencing |
| *gon4la-S3* | ACTCATATCAGAGTCTCGGT | sequencing |
| *gon4la-S4* | TTAGCTGATGTGTTGAAGCA | sequencing |
| *gon4la-S5* | TACCCAGAGCTACTTCCTAC | sequencing |
| *gon4la-S6* | TCTGAGTGCCTTCAAAAAGA | sequencing |
| *gon4la-S7* | TCGCCAACACCTTTATATGT | sequencing |
| *gon4la-S8* | CTTTCTGAATCCTCTGGGAG | sequencing |
| *gon4la-S9* | GACTCAAGCCTATTCTGGAG | sequencing |
| *gon4la-S10* | TAGCAGTGCAATGTTGGATA | sequencing |
| *gon4la-AS2* | CCGGCTGGATTTCATCTTC | sequencing |
| *gon4la-AS6* | CAGATGCCTGGTTATATGTTC | sequencing |
| *gon4la-AS9* | ACCTCATACACCTTCTCCAG | sequencing |
| *gon4la-AS10* | TCCGCTAACATATTTCTGCT | sequencing |
