# Supplementary material for: Zebrafish gon4la mutants recapitulate human GON4L-related growth disorders and reveal novel metabolic organs abnormalities
Source: Sci Rep. 2026 Apr 4;16:16357. doi: 10.1038/s41598-026-44674-3 (PMC13212936; doi:10.1038/s41598-026-44674-3)
Supplement: Supplementary file 4 — Supplementary Material 4 [file 41598_2026_44674_MOESM4_ESM.pdf]

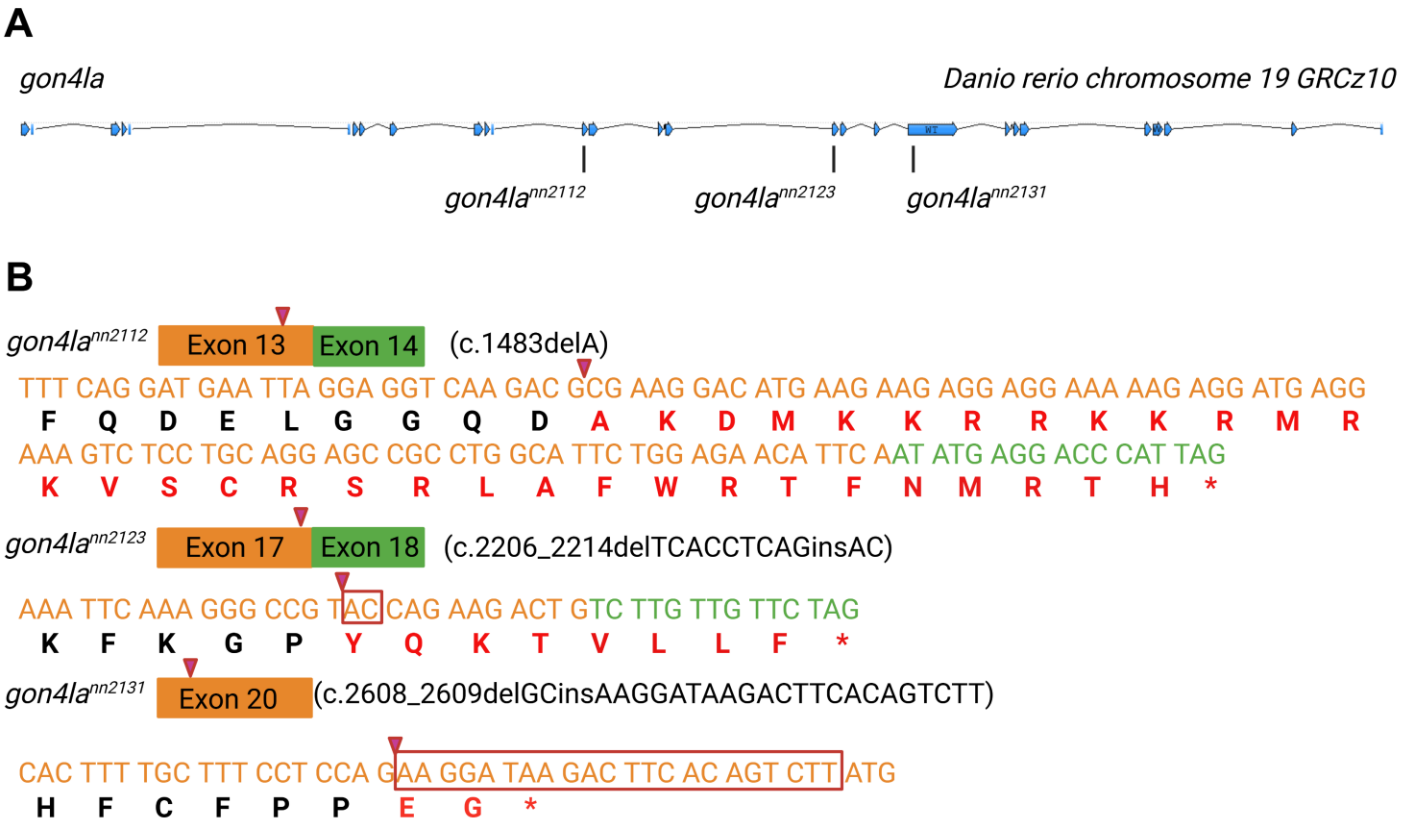

Supplementary Figure 1. Genomic structure and sequence validation of *gon4la* mutant alleles. (A) Schematic representation of the zebrafish *gon4la* genomic locus. The gene structure is shown with blue boxes representing exons (28 exons in total) and lines representing introns. The precise target sites for the three CRISPR/Cas9-generated alleles—*gon4la*^{nn2112}, *gon4la*^{nn2123}, and *gon4la*^{nn2131}—are indicated by vertical black bars, corresponding to exons 13, 17, and 20, respectively. (B) Nucleotide and predicted amino acid sequences of mutant alleles. For each allele, the specific cDNA mutation is provided in parentheses. Red arrowheads in the sequence alignment denote the exact position of the indel for each allele. Frameshift mutations and resulting premature stop codons (red asterisks) are shown for *nn2112* (exons 13/14; +32 novel a.a.), *nn2123* (exons 17/18; +8 novel a.a.), and *nn2131* (exon 20; +2 novel a.a.). Red boxed regions and red text highlight the altered nucleotide and predicted novel amino acid sequences resulting from the frameshift events. Orange and green highlights denote the respective exons involved in each frameshift event. cDNA sequencing of all three lines confirmed the retention of intended indels and the absence of alternative splicing or exon skipping that could restore the reading frame. These genomic details provide the molecular basis for the truncated Gon4la protein products observed in *in vitro* and *in vivo* assays. This figure is created with BioRender.com.

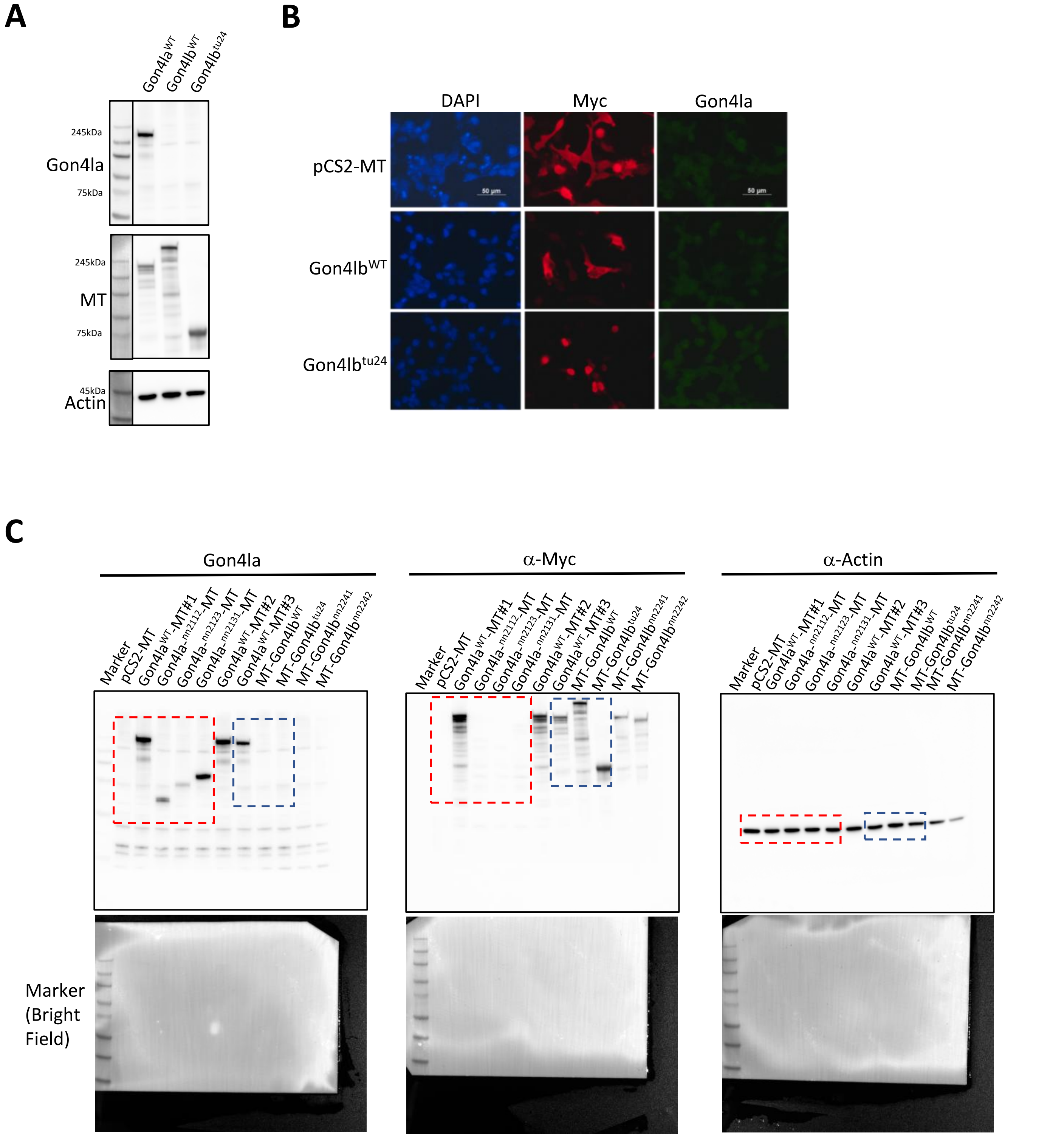

Supplementary Figure 2. Validation of Gon4la antibody specificity. To assess the specificity of the Gon4la antibody, N-terminal Myc-tagged Gon4lb constructs were transfected into cells and analyzed by (A) Western blot and (B) immunofluorescence staining. The Gon4la antibody did not cross-react with Gon4lb, confirming its specificity.(C)The original, uncropped images for all Western blots were provided. C-terminal Myc-tagged Gon4la and N-terminal Myc-tagged Gon4lb constructs were transfected for expression; for Gon4laWT-MT, #1–3 indicate independent biological replicates. Please note that the bands presented in Figure 1F (cropped from regions in red dashed boxes) and Supplementary Figure 2A (cropped from regions in blue dashed boxes) were cropped from the same original membrane/exposure to represent different experimental contexts. Bright field images showing molecular weight markers are included for verification. These original images confirm that no improper digital manipulation was performed.

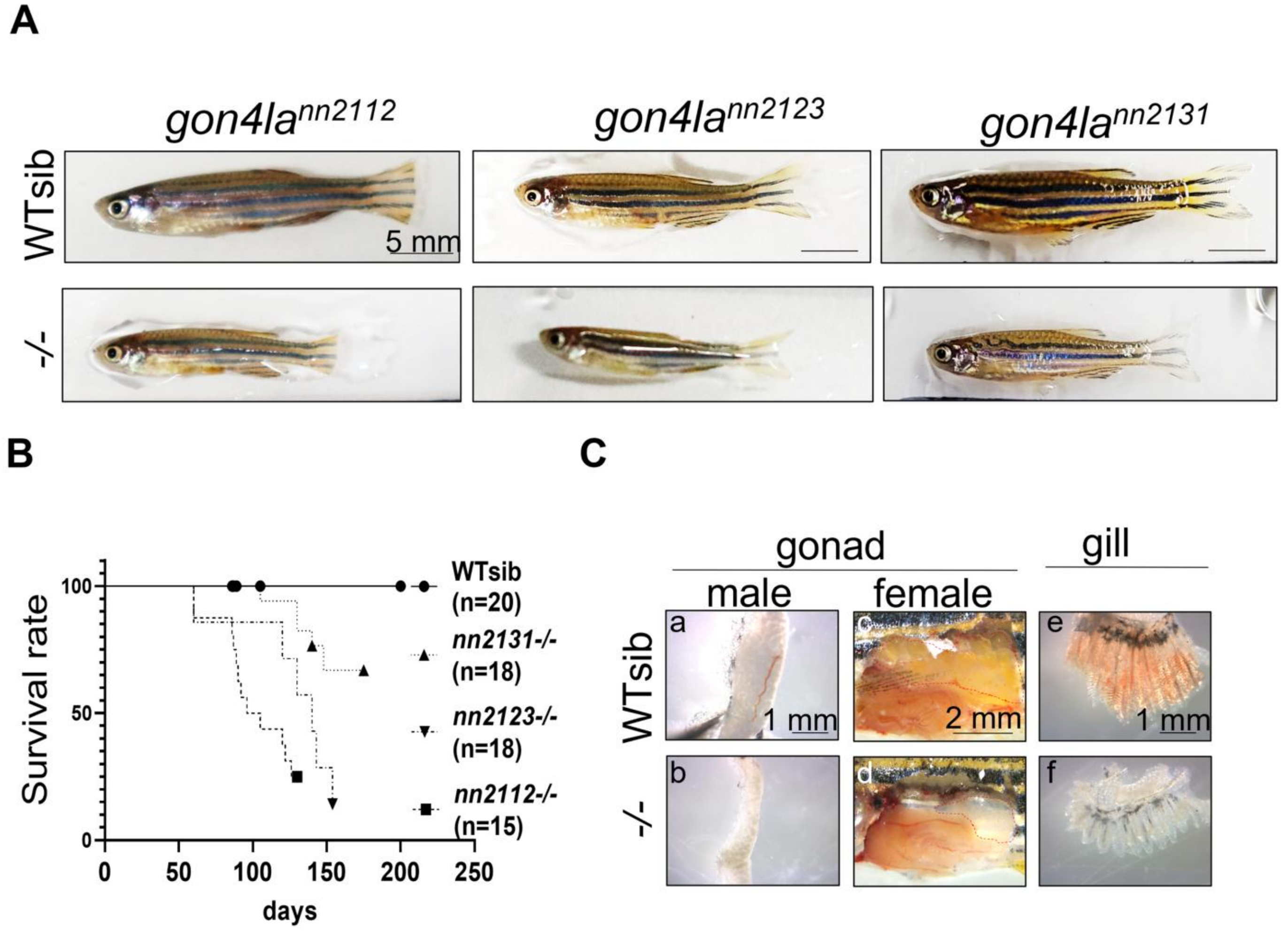

Supplementary Figure 3. Smaller stature and decreased survival rates are also observed in the *gon4la*^{nn2123} and *gon4la*^{nn2131} mutant lines. (A) Body size comparison between *gon4la* mutant fish lines and respective WT siblings at 3 months old. (B) Survival rates were reduced in all three *gon4la* mutant lines. Survival curves for WT (n=20), *gon4la*^{nn2112} (n=15), *gon4la*^{nn2123} (n=18), and *gon4la*^{nn2131} (n=18) homozygous mutant lines were monitored after genotyping at 1 month old. (C) Additional abnormalities in mutant fish, including underdeveloped gonads in males (a, b) and females (c, d), and bloodless gills in both sexes (e, f), observed at 3 months of age. Red dashed lines indicate the location of ovaries.

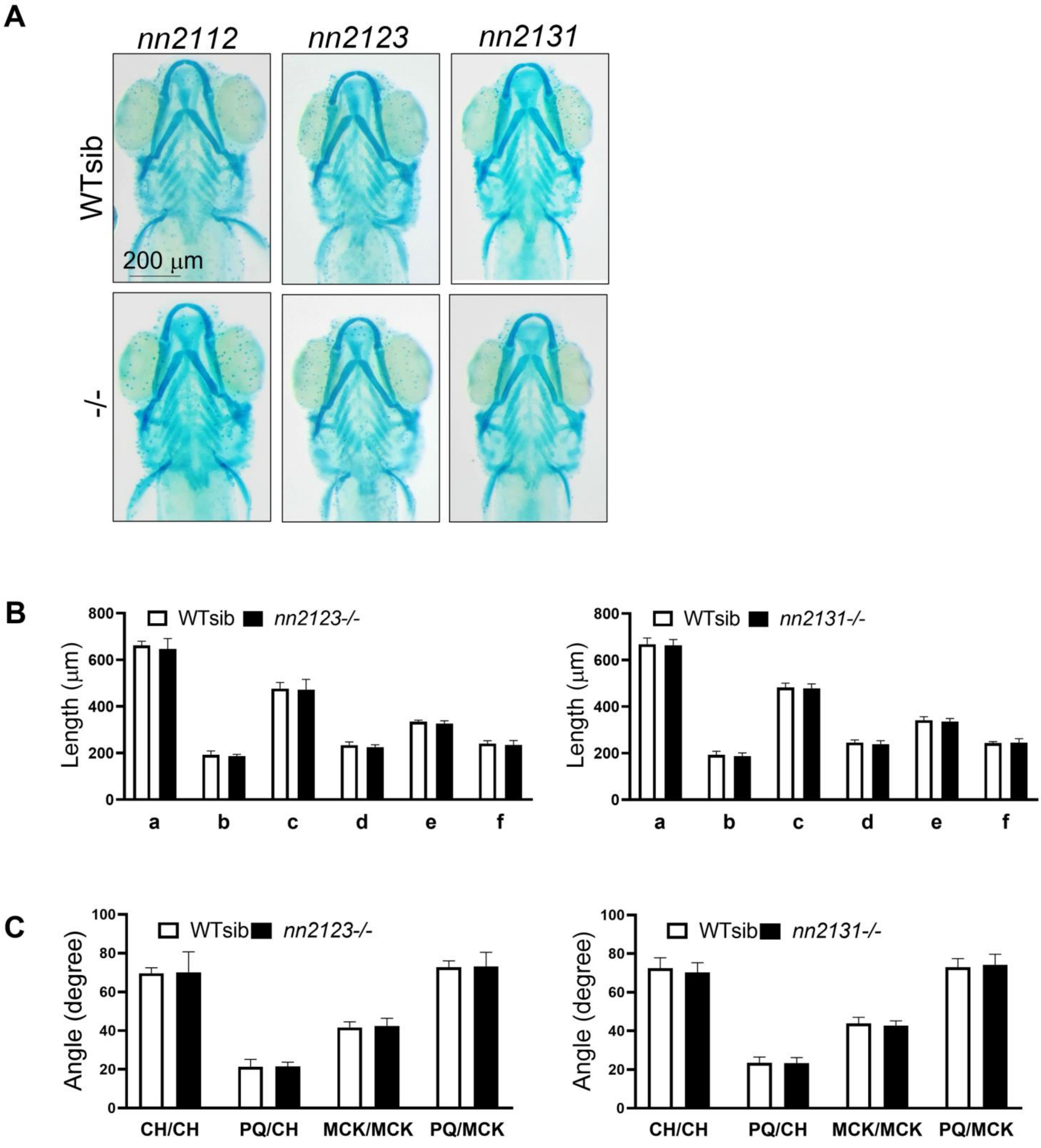

Supplementary Figure 4. Normal craniofacial development in *gon4la* mutant lines at 5 dpf. (A) Craniofacial bone structure of WT siblings and *gon4la*^{nn2123} and *gon4la*^{nn2131} mutant larvae stained with Alcian blue at 5 dpf. The craniofacial bones used for length and angle measurements are indicated in the legend of Figure 2G. (B) Quantification of cartilage lengths. (C) Measurement of four craniofacial angles. All angles were comparable across genotypes except for a reduction in the PQ/CH angle in *gon4la*^{nn2112} mutants (Fig. 2J). n = 9–10 per group. $p < 0.05$.

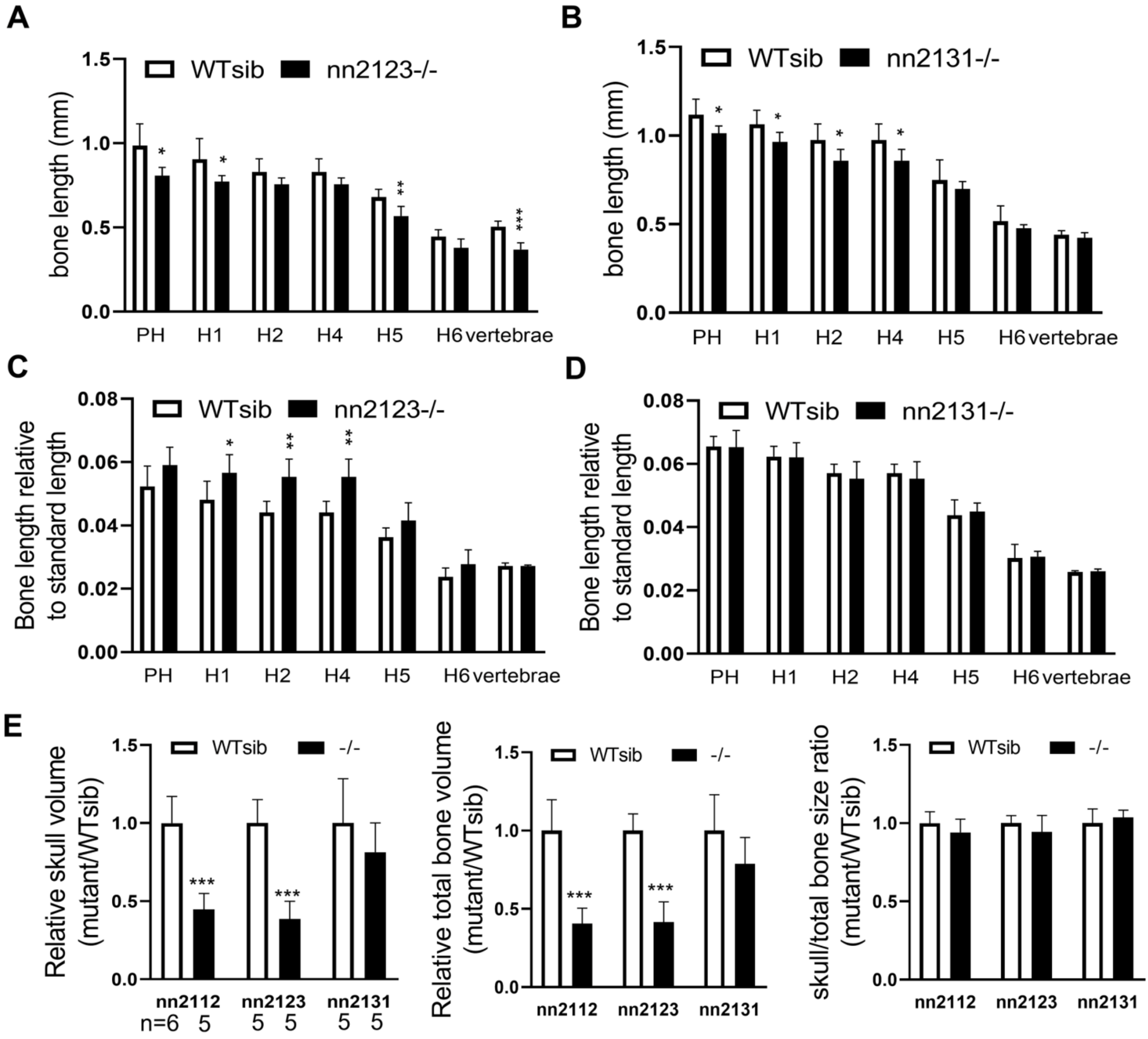

Supplementary Figure 5. Proportionate dwarfism phenotypes are also observed in the *gon4la*^{nn2123} and *gon4la*^{nn2131} mutant lines. Micro-CT images of the caudal fin and vertebrae in WT siblings and *gon4la* mutants at 3–4 months old were taken. (A, B) Lengths of endochondral bones, including the parhypural (PH) and hypurals (H1, H2, H4, H5, H6), as well as caudal vertebrae, were measured in *gon4la*^{nn2123} and *gon4la*^{nn2131} mutant lines after μCT imaging. n = 5 per group. (C, D) Ratios of bone lengths to standard length. (E) Relative skull volume, relative total bone volume (for mutant fish compared to WT siblings), and the skull-to-total bone volume ratio were determined for each genetic line. n = 5 per group. *, $p < 0.05$; **, $p < 0.01$; ***, $p < 0.001$.

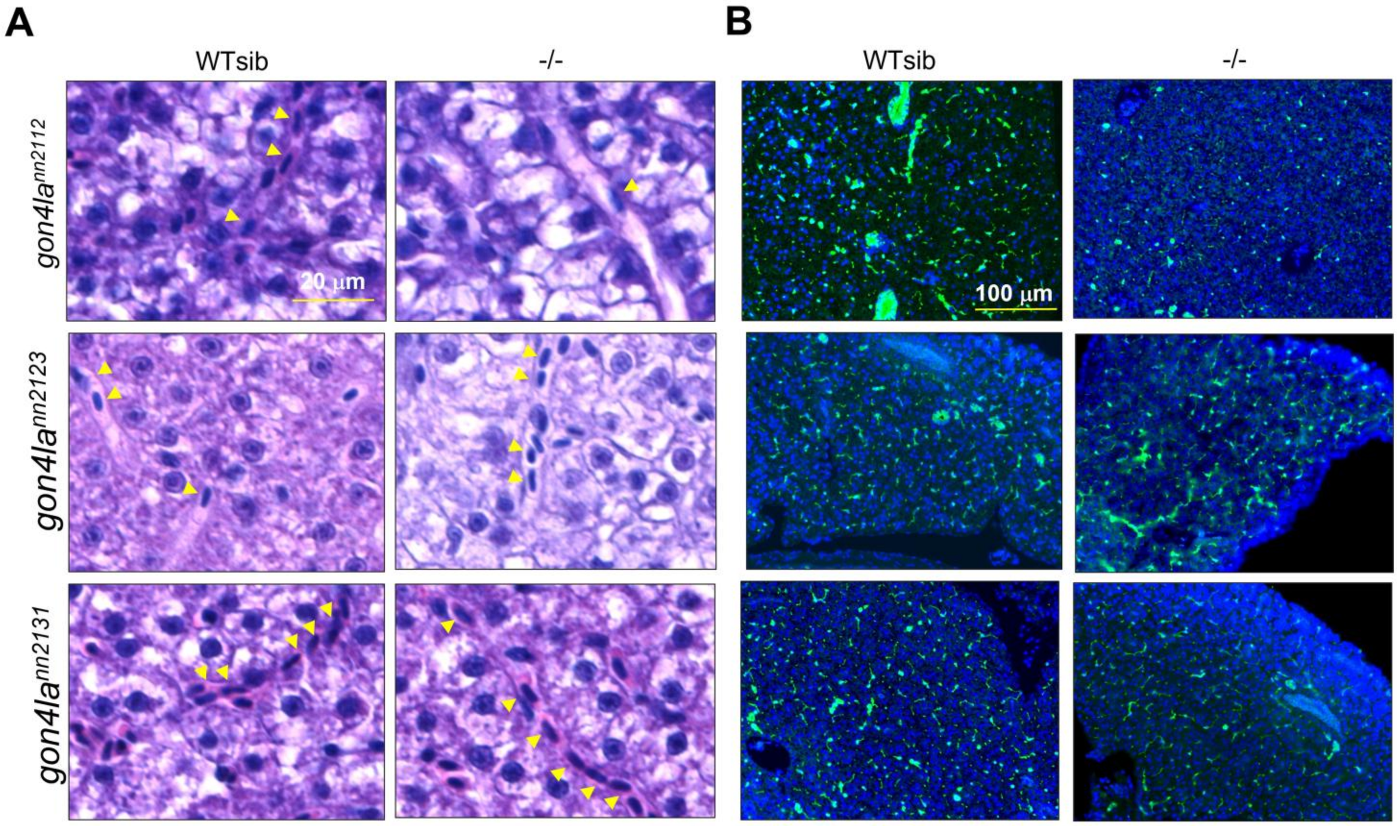

Supplementary Figure 6. Histological changes observed in livers of *gon4la*^{nn2112} mutant line. (A) Widened diameter of sinusoidal vasculatures in *gon4la*^{nn2112} mutant liver. Arrowheads indicate red blood cells within sinusoidal vasculatures. (B) Immunofluorescent staining of biliary epithelial cells (BECs). Liver sections stained with 2F11 antibody and AlexaFluor-488-conjugated secondary antibody show that fewer 2F11-positive BECs were observed in the liver of *gon4la*^{nn2112} mutant fish. Normal BEC differentiation was observed in livers of the other two *gon4la* mutant lines.

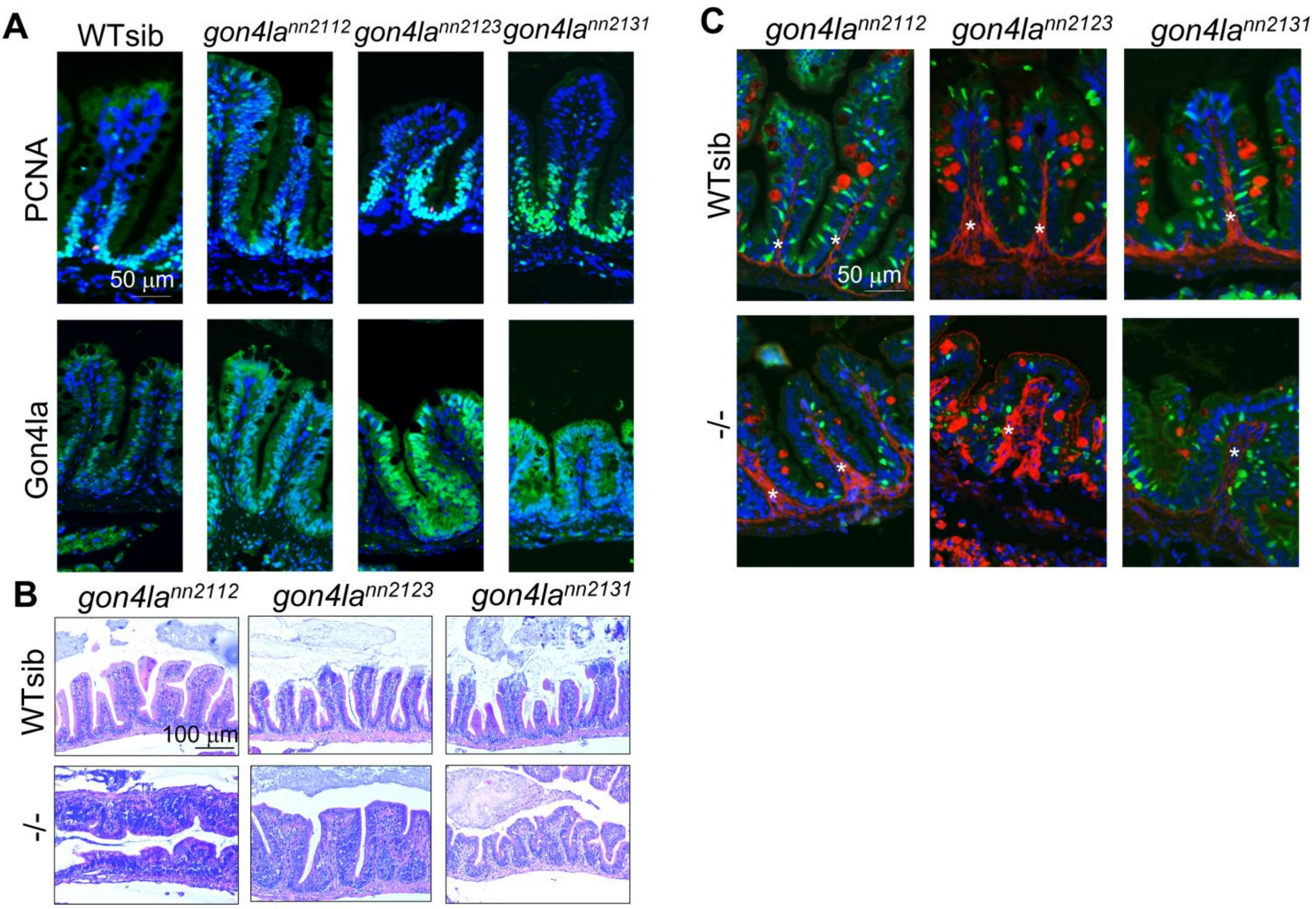

Supplementary Figure 7. Proliferation and differentiation of intestinal cells are dysregulated in all three *gon4la* mutant lines. (A) Immunofluorescent staining for PCNA (top) and Gon4la (bottom) reveals increased proliferation and expanded Gon4la expression in mutants (B) H&E staining of posterior intestine of WT siblings and *gon4la* mutant fish. (C) Immunofluorescent staining for goblet cells (WGA, red) and enteroendocrine cells (2F11, green). DAPI is used as a nuclear counterstain. Note the altered morphology of enteroendocrine cells and the depleted mucin within the theca of goblet cells in mutants. Asterisks indicate capillary WGA signal.

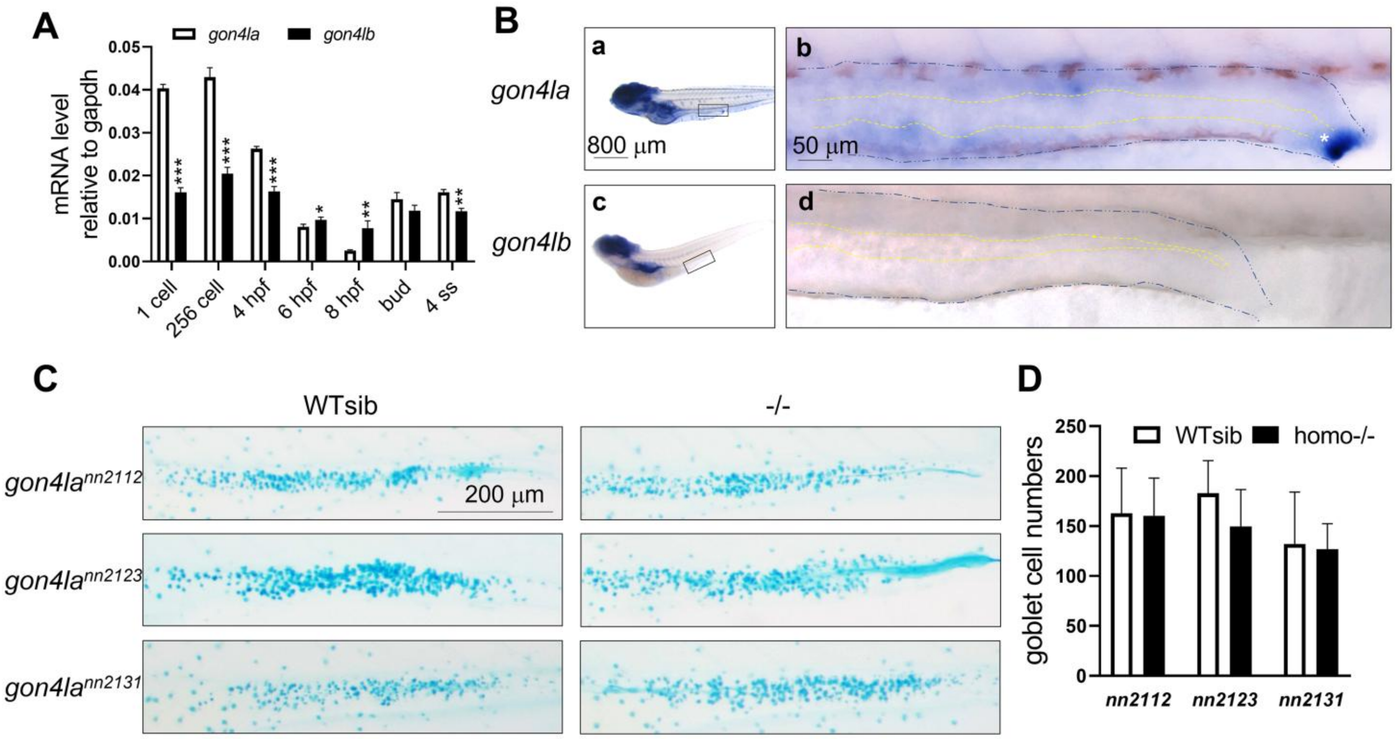

Supplementary Figure 8. Spatial divergence of *gon4la/b* expression and early intestinal secretory cell analysis. (A) qRT-PCR analysis of *gon4la* and *gon4lb* temporal expression. *gon4la* exhibits higher maternal expression levels compared to *gon4lb*. However, *gon4la* expression declines significantly around 6–8 hpf, a critical window during which *gon4lb* levels are predominant and essential for axis extension. (B) Whole-mount *in situ* hybridization for *gon4la* (a, b) and *gon4lb* (c, d). High-magnification views of the posterior intestine show that *gon4la* is expressed in posterior gut folds and cloaca (asterisk), *gon4lb* expression is absent or below the detection limit in these tissues. Blue dashed lines indicate gut margins; yellow dashed lines indicate lumen boundaries. This spatial segregation supports the model where *gon4lb* cannot provide compensatory support for *gon4la* during intestinal maturation. (C) Alcian blue staining for goblet cells in the intestine of WT and mutant larvae at 5 dpf. (D) Quantification of goblet cell numbers. No significant difference was observed between WT and mutants at this larval stage (n = 6-10 for each group), suggesting that the severe goblet cell depletion observed in adults (Fig. 5) is a progressive defect following the larval-to-adult transition.
